# Genetic Underpinnings of Carotenogenesis and Light-Induced Transcriptome Remodeling in the Opportunistic Pathogen *Mycobacterium kansasii*

**DOI:** 10.3390/pathogens12010086

**Published:** 2023-01-05

**Authors:** Niklas Janisch, Keith Levendosky, William C. Budell, Luis E. N. Quadri

**Affiliations:** 1 Department of Biology, Brooklyn College, City University of New York, 2900 Bedford Avenue, Brooklyn, NY 11210, USA; 2 Biology Program, Graduate Center, City University of New York, 365 Fifth Avenue, New York, NY 10016, USA; 3 Biochemistry Program, Graduate Center, City University of New York, 365 Fifth Avenue, New York, NY 10016, USA

**Keywords:** *Mycobacterium kansasii*, nontuberculous mycobacteria, carotene biosynthesis, pigment production, carotenoid cleavage oxygenase, light regulated genes, photolyase, MarR family, TetR/AcrR family, transcriptome, isoprenoid metabolism, photochromogenicity, oleic acid biosynthesis

## Abstract

*Mycobacterium kansasii* (*Mk*) causes opportunistic pulmonary infections with tuberculosis-like features. The bacterium is well known for its photochromogenicity, i.e., the production of carotenoid pigments in response to light. The genetics defining the photochromogenic phenotype of *Mk* has not been investigated and defined pigmentation mutants to facilitate studies on the role of carotenes in the bacterium’s biology are not available thus far. In this study, we set out to identify genetic determinants involved in *Mk* photochromogenicity. We screened a library of ~150,000 transposon mutants for colonies with pigmentation abnormalities. The screen rendered a collection of ~200 mutants. Each of these mutants could be assigned to one of four distinct phenotypic groups. The insertion sites in the mutant collection clustered in three chromosomal regions. A combination of phenotypic analysis, sequence bioinformatics, and gene expression studies linked these regions to carotene biosynthesis, carotene degradation, and monounsaturated fatty acid biosynthesis. Furthermore, introduction of the identified carotenoid biosynthetic gene cluster into non-pigmented *Mycobacterium smegmatis* endowed the bacterium with photochromogenicity. The studies also led to identification of MarR-type and TetR/AcrR-type regulators controlling photochromogenicity and carotenoid breakdown, respectively. Lastly, the work presented also provides a first insight into the *Mk* transcriptome changes in response to light.

## 1. Introduction

The opportunistic nontuberculous mycobacterial pathogen *Mycobacterium kansasii* (*Mk*) is the best known and most clinically significant species (also known as *Mk* subtype 1) of the recently proposed *Mk* complex [1,2]. *Mk* is a close relative of *Mycobacterium tuberculosis* (*Mt*) and causes life-threatening chronic pulmonary disease with tuberculosis-like features and mortality associated with treatment failure and comorbidities [3,4,5,6,7,8,9,10]. Most *Mk* lung infections are thought to be acquired through exposure to aerosolized environmental bacteria present in urban water systems, which represent a postulated major pathogen reservoir [11,12,13]. Skin and subcutaneous infections are not uncommon and, like primary lung infection, can disseminate in immunocompromised individuals [3,14,15,16,17]. *Mk* infections are difficult to control and eradicate and, paralleling the drug therapy for tuberculosis, they require costly, long-term multidrug courses with adverse side effects and problematic compliance [17,18,19,20,21].

*Mk* has become one of the six most common nontuberculous mycobacterial pathogens associated with chronic pulmonary disease in the United States and many other areas of the world, where it is often surpassed only by the *Mycobacterium avium* complex [21,22,23,24,25,26,27,28,29,30]. The global prevalence of *Mk* chronic pulmonary disease remains underrepresented, particularly in high tuberculosis/HIV-AIDS burden areas of the world, owing to overlapping features with tuberculosis, misdiagnoses, lack of compulsory reporting policies, and the recent disruptions to the global healthcare infrastructure during the COVID-19 pandemic [31,32,33].

Despite the burden of *Mk* infections, the bacterium’s biology has remained poorly explored. In particular, the scarcity of gene function studies and the lack of genetically defined mutants are noticeable. To our knowledge, there are only a handful of reported studies of directed genetic manipulation of *Mk* to study gene function [34,35,36,37,38]. This narrow record is, in part, due to a relatively limited availability of validated tools for straightforward genetic manipulation of *Mk*. We recently demonstrated the functionality of the powerful φMycoMarT7 transposon (Tn) mutagenesis system in *Mk* [38], thus expanding the repertoire of genetic manipulation tools available to accelerate the dissection of gene function and the generation of gene knockouts in this recalcitrant mycobacterial pathogen.

One of the well documented and characteristic phenotypic traits of *Mk*, a bacterium early on referred to as a “*yellow bacillus*” [39], is its photochromogenicity, i.e., the ability to produce carotenoid pigments in response to light [40]. *Mk* colonies and cells in liquid cultures are essentially devoid of pigmentation (off-white color) when grown in the dark, but develop an intense yellow pigmentation upon exposure to light. This light-induced pigmentation is due to the accumulation of carotenoid compounds, primarily β-carotene [41]. To our knowledge, the genetics of *Mk* photochromogenicity has not been investigated as of yet, and there are no defined carotenoid production-deficient mutants available for use in research aimed at investigating the roles of pigments in the biology of the bacterium. In this study, we harnessed the power of large-scale Tn mutagenesis-based forward genetic screening and gene expression profiling analysis to illuminate the genetic underpinnings of the photochromogenic phenotype of *Mk* and provide a first insight into the transcriptome changes induced by light in this opportunistic pathogen. In all, our findings provide novel information on the biology of *Mk* that (i) expands our understanding of carotenoid biosynthesis and its light-dependent regulation, (ii) illuminates a carotenoid degradation pathway and its light-independent control, and (iii) demonstrates the presence of light-induced genes beyond those involved in photochromogenicity. In addition, the collection of novel *Mk* mutants generated in this study will facilitate future research aimed at investigating the relevance of carotenoids in the general biology and virulence of the pathogen.

## 2. Results and Discussion

### 2.1. The Screen of an M. kansasii Transposon Library for Pigmentation Mutants Led to Identification of Four Distinct Mutant Phenotype Groups

To begin a dissection of the genetic determinants involved in *Mk* photochromogenicity, we carried out a large-scale screen for mutants with pigmentation abnormalities in a library of ~150,000 Tn insertion mutants. The *Mk* mutant library was generated using the φMycoMarT7 phage-based transduction method [42], which we have recently validated for use in *Mk* [38]. The method delivers a mariner Tn, which essentially inserts randomly at TA dinucleotides (TA sites). As per our analysis of the annotated *Mk* genome, the bacterium has 99,953 TA sites (97,702 in the chromosome and 2251 in the pMK12478 plasmid), one TA site per 65.7 nucleotides on average, and an average gene size of ~1 kb with 15 TA sites. The screened library corresponded to a 1.5-fold coverage relative to the number of TA sites in the genome. Of note, there are 19 annotated small genes (out of ~5550) in *Mk* that lack TA sites (all chromosomal), and thus these genes cannot be disrupted by the Tn. However, insertion data of *cis*-acting extragenic regions (e.g., promoters) or 5′ untranslated regions (5′-UTRs) might provide insight into the potential involvement of some of these genes in photochromogenicity. There are also 77 genes and 136 genes with a single and two TA sites, respectively. The 1.5-fold coverage of our library brings the theoretical probability of the Tn missing these most challenging genes by chance to 0.2 and 0.05, respectively (Figure S1).

**Figure 1:**
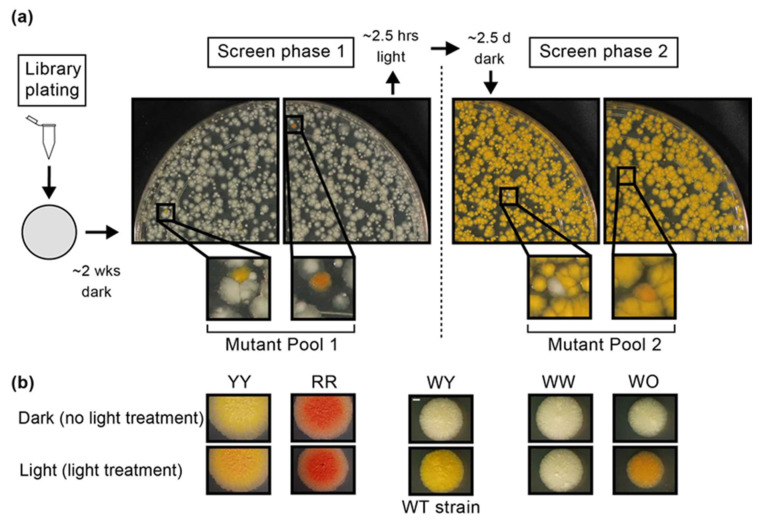
Screen for pigmentation mutants of *M. kansasii*. (**a**) Plates were screened for pigmentation mutants after incubation for colony development in the dark (~14 d), and the identified mutants (mutant pool 1) were recovered from the plates (screen phase 1). The screened plates were then exposed to light (~2.5 h) for pigment induction, and subsequently incubated in the dark (~2.5 d) for colony pigmentation development. After incubation, plates were re-screened for pigmentation mutants and the identified mutants (mutant pool 2) were isolated from the plates (screen phase 2). Representative images of screen plates’ quadrants and enlarged (4×) plates’ excerpts highlighting the four types of pigmentation mutants identified in the screen are shown. Left and right excerpts of screen phase 1 show yellow and red mutants, respectively, surrounded by wild-type (WT)-looking white colonies. Left and right excerpts of screen phase 2 show white and orange mutants, respectively, surrounded by WT-looking yellow colonies. Before light exposure, the orange mutant was white, as were the surrounding colonies (not shown). Screen plate size = 15 cm diameter. (**b**) Macrocolonies (arising from spot-inoculation of liquid cultures) of mutants representative of the four phenotypic groups identified in the mutant pools 1 and 2. Pool 1 included YY (yellow in dark and light) and RR (red in dark and light) mutants, whereas pool 2 was composed of WW (white in dark and light) and WO (white in dark and orange in light) mutants. The WT strain (center column; WY phenotype, i.e., white in dark and yellow in light) is included as reference. Macrocolonies were grown in the dark for ~14 d without (‘Dark’ row) or with a ~2.5 h light treatment at day 12 (‘Light’ row). White scale bar = 2 mm.

We carried out the screening of the entire library for mutant colonies with pigmentation abnormalities in two phases, one with a screen before light treatment and a second one with a screen after light treatment (Figure 1a). In the first phase, plates with colonies grown in the dark were screened (under normal laboratory illumination) for mutants with a coloration clearly distinguishable from the wild-type (WT) off-white appearance of the rest of the colonies on the plates. After this screen, all plates were equally exposed to white fluorescent light to homogeneously trigger carotenoid pigment production across all plates. After the light treatment, the plates were returned to incubation to allow for the development of the typical light-induced yellow coloration of *Mk* colonies, and then screened for a second time for colonies with pigmentation characteristics diverging from the WT yellow coloration of the rest of the colonies on the plates. All pigmentation mutants identified in the screens were recovered from the plates and subjected to colony purification and pigmentation phenotype verification with and without light treatment. Mutant isolates that did not show a clearly reliable mutant pigmentation phenotype were not investigated further.

Our first screen phase led to the isolation of 83 mutants with clearly recognizable pigmentation abnormalities deviating from the WT phenotype, i.e., off-white in the dark, but yellow when exposed to light (a phenotype hereafter referred to as WY). Each of these 83 mutants could be assigned to one of two phenotypic groups. The mutants displayed a yellow color prior to and after light treatment (hereafter referred to as YY phenotype; 58 mutants, 70%) or had an anomalous reddish color prior to and after light treatment (hereafter referred to as RR phenotype; 25 mutants, 30%) (Figure 1b). The second screen phase rendered 121 mutants with robust pigmentation oddities. Most of these mutants failed to develop pigmentation upon light treatment and retained an off-white appearance (a phenotype hereafter referred to as WW; 98 mutants, 81%). The remaining mutants transitioned from the off-white appearance to an abnormal orange color after light treatment (a phenotype hereafter referred to as WO; 23 mutants, 19%) (Figure 1b). In all, our screen rendered a collection of 204 pigmentation mutants (0.14% mutant isolation rate) distributed across four phenotypic groups clearly distinguishable from each other and from the WT phenotype.

### 2.2. The Insertion Sites in the Pigmentation Mutant Collection Clustered in Three Chromosomal Regions

**Figure 2:**
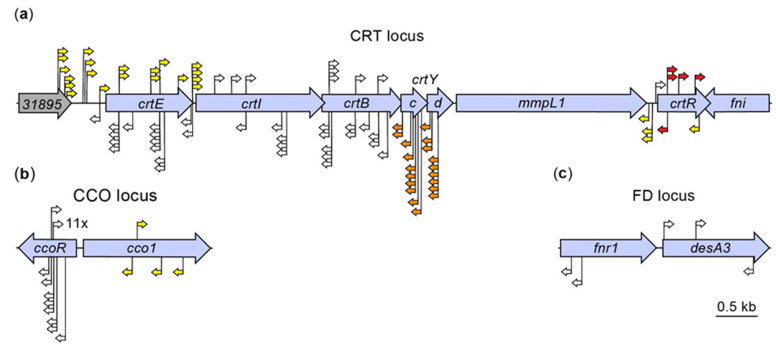
Genomic locations of transposon insertions in the pigmentation mutants. (**a**) Insertions in the carotenoid biosynthesis (CRT) locus. The number label of the gene at the 5′ end corresponds to the MKAN_RS locus tag. (**b**) Insertions in the carotenoid cleavage oxygenase (CCO) locus. The 11× notation signifies that 11 mutants with the same insertion site and Tn orientation were found at the location. (**c**) Insertions in the *fnr1-desA3* (FD) locus. Each yellow, white, orange, or red arrow marks the position of the Tn and the orientation of the Tn’s Km^R^ gene in a particular mutant. Overlapping arrows indicate insertions at the same TA site. The color of the arrow denotes the mutant’s phenotypic group. White, yellow, red, and orange arrows signify WW; YY, RR, and WO groups, respectively. Genomic coordinates of the segments displayed: (**a**), 2342688–2351750; (**b**), 4262321–4265776; (**c**), 4704860–4707354. Information on precise insertion sites, gene locus tags, and predicted gene functions is provided in Table S1.

We determined the location of the insertion for 124 of our 204 mutants. The 124 mutants encompassed 31 (53%), 5 (20%), 69 (70%) and 19 (83%) of the isolates with YY, RR, WW and WO phenotype, respectively. The results of the insertion site determination are summarized in Table S1. We found that all insertions were located in the chromosome, with 116 (94%) of them mapping to three chromosomal segments with multiple insertions each (Figure 2a–c). These segments are hereafter referred to as the carotenoid biosynthesis (CRT), the carotenoid cleavage oxygenase (CCO), and the *fnr1-desA3* (FD) loci. These loci are discussed in ensuing sections. The genes disrupted in these loci added up to eleven, a number representing ~0.2% of the annotated *Mk* genes. The remaining eight mutants mapped to eight loci scattered across the chromosome (Table S1). Our sequence bioinformatics and literature analysis did not reveal any likely connection between these loci and the pigmentation phenotypes of the eight mutants (5 WW and 3 YY). We suspect that the phenotype of each of these mutants is unrelated to the mapped insertion, but likely resulted from a spontaneous mutation (or an unmapped secondary Tn insertion) in the CRT, CCO, or FD loci. These mutants were not further investigated or discussed herein.

### 2.3. Sequence Bioinformatics and Insertional Analysis of the Carotenoid Biosynthesis Locus

**Figure 3:**
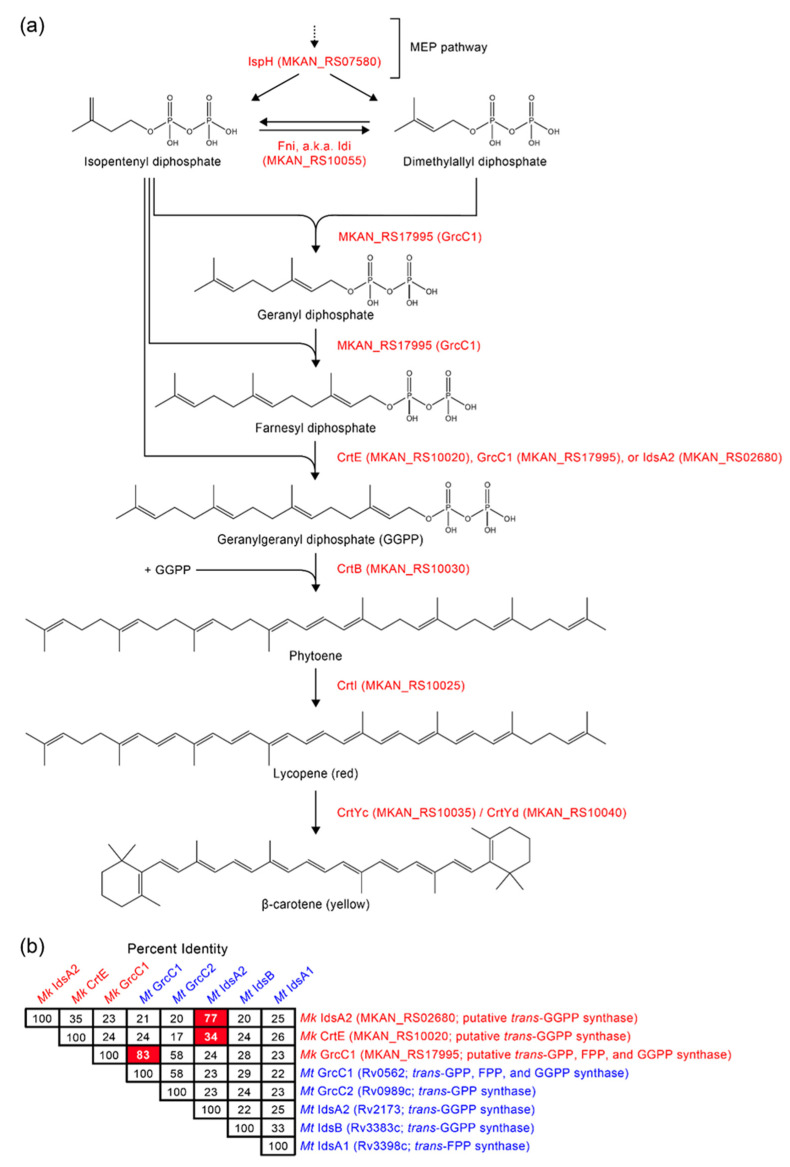
Proposed carotenoid biosynthesis pathway of *M. kansasii*. (**a**) The scheme highlights the functions of the *M. kansasii* enzymes encoded by the genes described in the presented work, in the context of the general bacterial carotenogenesis pathway [45]. Synthesis of geranylgeranyl diphosphate might take place via the predicted geranylgeranyl diphosphate synthase CrtE or by alternative CrtE-independent routes with putative *trans*-isoprenyl diphosphate synthases we identified by sequence analysis. The final pathway product shown is β-carotene, the major carotenoid produced by *M. kansasii*. (**b**) Pairwise amino acid sequence identity matrix for predicted *trans*-isoprenyl diphosphate synthases of *M. kansasii* (*Mk*, names in red) and *M. tuberculosis* (*Mt*, names in blue). Proteins are labeled with their locus tags and assigned or herein proposed protein names for *Mt* and *Mk*, respectively. Amino acid identity percentages of the best *Mk*-*Mt* pairs are highlighted (red boxes). Sequence alignment was done with the Clustal W algorithm embedded in the MegAlign application of the DNASTAR Lasergene software.

We identified 87 insertions mapping to a multigene locus encoding predicted carotenoid biosynthesis enzymes (Figure 2a). Of these insertions, 72 were distributed across 7 protein-coding genes, whereas the remaining 15 mapped to intergenic/promoter regions or 5′-UTRs. Our sequence analysis of the CRT locus-containing fragment indicated that 5 genes (hereafter referred to as *crtE*, *crtI*, *crtB*, *crtYc*, and *crtYd*) out of the 7 had an operon-like organization and encoded putative orthologues of the enzymes geranylgeranyl diphosphate synthase (*crtE*), phytoene desaturase (*crtI*), phytoene synthase (*crtB*), and heterodimeric lycopene cyclase (*crtYc* and *crtYd*) involved in synthesis of β-carotene in bacteria [43] (Table S1). Based on these findings and additional analysis described in ensuing text, we propose the involvement of these and other *Mk* genes discussed below in the *Mk* carotene biosynthesis pathway outlined in Figure 3a. In addition, several insertions mapped to the very 3′ end of *MKAN_RS31895* (formerly annotated as *MKAN_RS31470* and *MKAN_RS29525*), which is located immediately upstream of *crtE* and encodes a predicted glycoside hydrolase of unknown function (Figure 2a). However, our orthology and literature analyses did not uncover any potential link between *MKAN_RS31895* and carotenogenesis. Lastly, the seventh gene we identified with insertions encodes a putative MarR family regulator with unknown function (hereafter referred to as *crtR*). Interestingly, almost two decades ago, Gao et al. reported that a Tn mutant of the *crtR* orthologue in the photochromogenic *Mycobacterium marinum* displayed constitutive pigment production, yet the involvement of the gene in carotenogenesis was not conclusively established [44].

**Figure 4:**
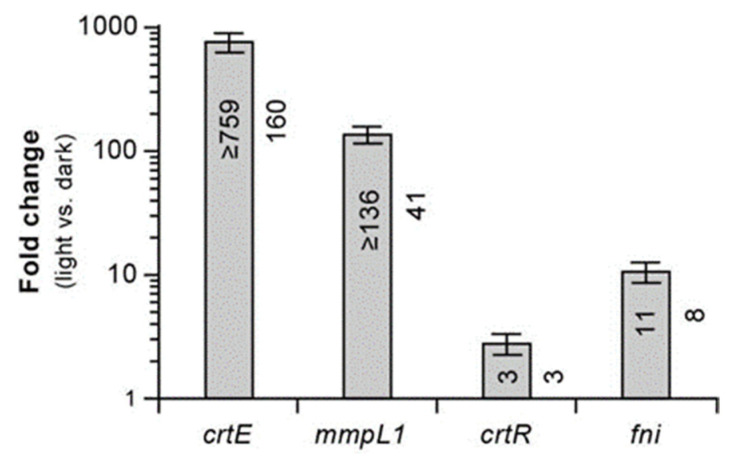
RT-qPCR analysis of the effect of light exposure on the expression of CRT locus genes in wild-type *M. kansasii*. The values in the bars indicate expression fold changes in light-treated cultures relative to untreated cultures. Strand specific RT-qPCR was used for the determinations. Data represent means ± SEM of three independent biological replicates. The fold change values are preceded by the ≥ symbol when the gene expression values in the untreated (dark condition) culture were at or below the limit of detection. The values outside the bars are the expression fold changes determined by RNA-seq analysis (See below).

Notably, our exploration of the chromosome region downstream of *crtR* revealed the presence of a putative isopentenyl diphosphate (IPP) delta-isomerase gene (*fni*, also known as *idi, MKAN_RS10055*) located immediately adjacent to and convergently oriented with the regulator gene (Figure 2a). IPP isomerases interconvert IPP and dimethylallyl diphosphate (DMAPP) [46], two precursors for biosynthesis of carotenoids (Figure 3a). Both IPP and DMAPP are products of the 2C-methyl-D-erythritol 4-phosphate (MEP) pathway [47] (Figure 3a), an essential pathway for synthesis of isoprenoid precursors and growth in mycobacteria [45]. The *fni* gene is not essential in *Mk* [38], and its 13 TA sites make the probability of being missed by the Tn by chance ~10^−9^ (Figure S1). Thus, we conclude that the absence of *fni* Tn mutants in the group of isolates analyzed is unlikely due to chance, and it indicates that *fni* disruption does not lead to abnormal pigmentation. IPP isomerases are thought to balance the pools of IPP and DMAPP [46], and thus our results suggest that Fni-dependent balancing of these precursor pools is not critical for carotenoid biosynthesis in *Mk*. We note, however, that the essentiality studies cited were not done under the strict dark conditions used in phase 1 of our screen. Thus, we cannot rule out the possibility that *fni* and other genes we indicate in the ensuing text to be nonessential as per previous studies are essential under the strict dark conditions used herein. It is noteworthy that, although we did not identify insertions in *fni*, the gene was found to be co-regulated by light along with other genes in the CRT locus, as per RT-qPCR (Figure 4) and RNA-seq analysis described below (Table 1, Figure S2a,b).

We also did not identify insertion mutants for a large gene (*MKAN_RS10045*; hereafter referred to as *mmpL1*) encoding an MmpL family protein member and located between the *crtEIBYcYd* cluster and *crtR* (Figure 2a). We have established that *mmpL1* is not essential in *Mk* [38], and the probability of the Tn missing *mmpL1* (with 37 TA sites) by chance is ~10^−25^ (Figure S1). Thus, we conclude that the lack of *mmpL1* Tn mutants in the collection of isolates analyzed indicates that insertions in this gene have no impact on pigmentation. MmpL transporters are involved in export of complex lipids of the mycobacterial cell envelope and siderophores across the plasma membrane [48]. There are 15 genes (all chromosomal) encoding MmpL proteins in *Mk* (Figure S3). It is tempting to hypothesize that *mmpL1* and at least one of its paralogues might have overlapping roles in transport of carotenoids, a functional redundancy that would prevent identification of *mmpL1* mutants in our screen. Alternatively, *mmpL1* might be essential for pigment transport, but pigment mislocalization in *mmpL1* mutants does not impact the pigmentation phenotype of the colonies under the experimental conditions of our screen.

**Figure 5:**
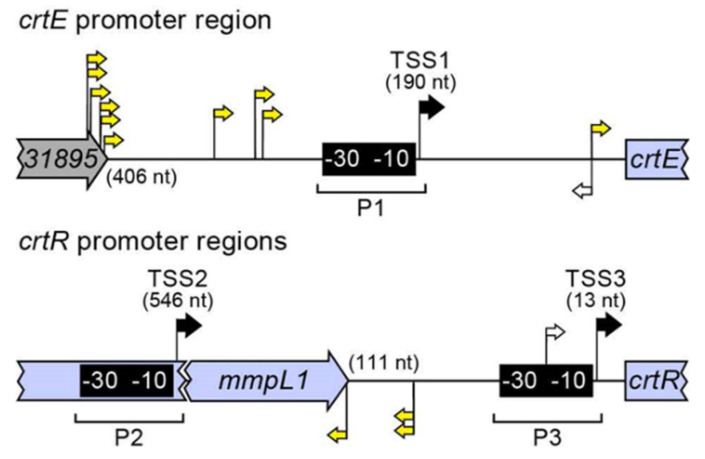
Insertions in the *crtE* (top scheme) and *crtR* (bottom scheme) promoter regions. Arrows demarcating insertions are as noted in Figure 2. The transcription start sites (TSS; solid black arrows) were determined by RNA-seq analysis (Figure S2a,b). The number of nucleotides (nt) between each TSS and the start of the downstream gene is indicated in parentheses. The locations of the putative −30 and −10 promoter elements depicted were predicted using the positions of their cognate TSS. The size of the entire intergenic region is given in parentheses at the 5′ end of the region.

Of the four pigmentation phenotypes seen in the screen isolates, the RR phenotype was the most striking. The phenotype was found only in mutants with insertions in the coding region of *crtR*, and it clearly contrasted with the YY or WW phenotypes produced by insertions in the intergenic region upstream of the gene (Figure 2a and Figure 5). Five of the six *crtR* mutants identified displayed the RR phenotype. This phenotype might suggest an abnormal accumulation of the red pathway intermediate lycopene irrespective of light conditions, a phenomenon that would emerge if lycopene cyclization were a rate-limiting step in the pathway to carotenes (Figure 3a). Notably, growing *Mk* under continuous light exposure produced a pigmentation phenotype consistent with lycopene accumulation and lycopene cyclization being rate-limiting under constant system photoinduction (Figure S4).

**Figure 6:**
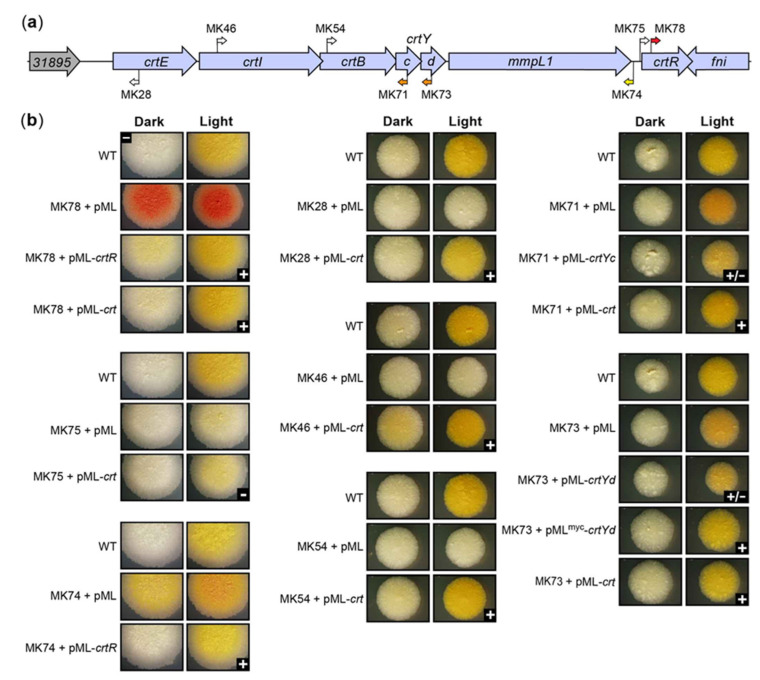
Macrocolony phenotype of selected CRT locus mutants and their complementation control strains. (**a**) CRT locus diagram. The insertions in the representative mutants analyzed are depicted. Arrows demarcating insertions are as noted in Figure 2. (**b**) Macrocolony phenotype. Macrocolonies were grown on two replicate plates in the dark (~2 wks). Then, one replicate was kept in the dark (‘Dark’ column) and the other exposed to light (~3 h) (‘Light’ column). Both replicates were subsequently grown in the dark (2–3 d) and imaged. WT, wild-type strain. pML, empty pML1335 vector. The mutant complementation control strains carried the indicated pML1335 derivatives expressing specific mycobacterial genes (Table S2). Symbols in the lower right corner of the images indicate complementation status: +, complete (or nearly complete) complementation; +/−, partial complementation; and −, no complementation. White scale bar (top left corner image) = 2 mm. The results shown are representative of three independent experiments.

We selected *crtR* mutant MK78 (Figure 6a) as representative of the five RR phenotype mutants for complementation analysis (Figure 6b). The *crtR* mutant MK78 with the RR phenotype was almost fully complemented by the WT gene expressed from its light-inducible promoter region (P3) located in the *mmpL1-crtR* intergenic region (P3-*crtR* fragment; Figure 5), which we identified by promoter prediction and the RNA-seq analysis of light-regulated genes described below (Figure S2b). The complemented strain regained the WT yellow color with light exposure, but displayed a slight yellow tint in the dark distinguishable from the off-white appearance of the WT strain (Figure 6b). However, transformation of MK78 with the 7.8 kb CRT locus fragment (i.e., *crtEIBYcYd*-*mmpL1*-*crtR*) led to full restoration of the WT WY phenotype (Figure 6b). Based on the results of these complementation tests and RNA-seq analysis described below suggesting that expression of *crtR* is also driven by promoters (P1 and P2, Figure 5) upstream the *mmpL1-crtR* intergenic region (Figure S2a,b), we attribute the complementation phenotype when only the P3-*crtR* fragment is introduced into MK78 to insufficient *crtR* expression.

**Figure 7:**
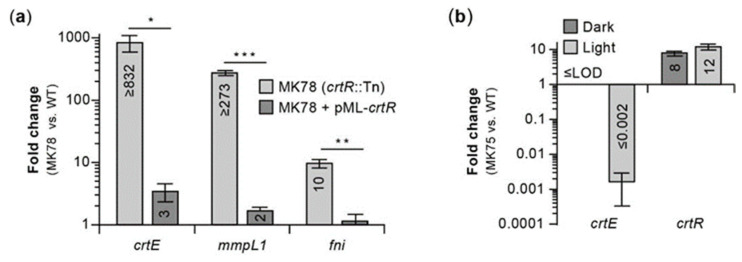
(**a**) RT-qPCR analysis of the effect of *crtR* disruption on the expression of CRT locus genes. The values in the bars indicate expression fold changes in the *crtR* mutant MK78 (Figure 6a) or its complementation control strain relative to the wild-type strain (WT). The cultures were grown in the dark. The fold change values are preceded by the ≥ symbol if the gene-specific expression in the WT was at or below the limit of detection (LOD). Strand specific RT-qPCR was used for the determinations. Statistical significance was determined using an unpaired, two-tailed *t*-test with the Holm–Sidak method in GraphPad PRISM 6 (*, *p* < 0.05; **, *p* < 0.006; ***, *p* < 0.0005). (**b**) RT-qPCR analysis of the effect of Tn-driven constitutive *crtR* overexpression on *crtE* expression. The values in the bars indicate expression fold changes in the *crtR* mutant MK75 (Figure 6a) relative to the WT. Cultures were grown in the dark (dark gray) or in the light (light gray). Strand specific RT-qPCR was used for the determinations. The *crtE* fold change value of the experiment in the dark is noted as ≤LOD because gene expression was below LOD in both MK75 and WT. The *crtE* fold change value of the experiment in the light is preceded by the ≤ symbol because *crtE* expression in MK75 was at or below LOD. Data in (**a**) and (**b**) represent means ± SEM of three biological replicates.

The phenotype of the RR *crtR* mutants suggested to us that CrtR functions as a repressor of genes required for pigment production, exerting a particularly strong repression in the absence of light exposure, and essentially leading to pigment production shutdown in the dark. To test the predicted repressor function of CrtR, we carried out RT-qPCR analysis comparing the expression of *crtE*, *mmpL1*, and *fni* (which are upregulated by light exposure; Figure 4, Table 1, Figure S2a,b) in the *crtR* mutant MK78, MK78 complemented with the P3-*crtR* fragment, and the WT strain (Figure 7a). The resulting data revealed that disruption of *crtR* in MK78 led to a drastic upregulation of the three genes relative to the WT strain. In contrast, the expression levels in the complemented strain were only slightly increased, an outcome aligned with the nearly full phenotypic complementation described above for MK78 transformed with the P3-*crtR* fragment. Overall, based on these findings and the phenotypes of the *crtR* mutants, we postulate that CrtR indeed functions as a repressor of carotenogenesis genes in the absence of light, a role making CrtR a key regulator controlling the photochromogenic phenotype of *Mk*.

Interestingly, the sixth *crtR* mutant (MK82, with the Tn closest to the 3′ end of the gene) exhibited YY phenotype (Figure 2a), a trait consistent with accumulation of β-carotene irrespective of light conditions. The intensities of the yellow in MK82 and other YY mutants of the CRT locus under dark and light conditions were comparable to each other and to the light-induced yellow pigmentation seen in the WT strain. We reason that the YY phenotype of MK82 might indicate that the insertion close to the 3′ end of *crtR* leads to a partially active repressor without sufficient activity to fully suppress pigment production in the dark, but with enough activity to prevent the RR phenotype seen in MK78 and other RR *crtR* knockout mutants (Figure 2a).

Four mutants with insertions in the *mmpL1-crtR* intergenic region, which includes one of the light-inducible promoters driving *crtR* expression (i.e., P3; Figure 5 and Figure S2b), displayed either WW or YY phenotype, depending on Tn orientation (Figure 2a). Mutant MK75 (Figure 6a), with the insertion ~10 bp upstream the transcription start site of promoter P3 (TSS3) and the Tn inserted so that its Km^R^ marker and *crtR* were in the same orientation (Tn-gene co-orientation), showed WW phenotype. We could not complement MK75 with the P3-*crtR* fragment (not shown) or the *crtEIBYcYd*-*mmpL1*-*crtR* fragment (Figure 6b). The latter result is consistent with the idea that the Tn insertion, rather than spontaneous mutations in carotene biosynthetic genes, caused the WW phenotype in the mutant. Based on our conclusion that CrtR is a repressor of pigment production and the uncomplementable nature of MK75, we reasoned that the insertion in the mutant might have led to a Tn-driven overexpression of CrtR high enough to alter the regulatory balance in such a way that the carotenogenesis genes remained repressed under light exposure. To probe this idea, we carried out comparative RT-qPCR expression analysis between MK75 and the WT strain. The expression data demonstrated that Tn-driven expression of *crtR* led to a ~10-fold upregulation of the repressor gene relative to WT with or without light exposure (Figure 7b). Concomitantly, the expression of *crtE* (used as a reporter of CrtR-regulated genes) remained undetected in the dark condition (a result consistently seen for the WT strain) and was downregulated by at least 500-fold relative to WT under light exposure (Figure 7b). Based on these findings, we conclude that Tn-driven overexpression of *crtR* in MK75 resulted in a drastic downregulation (shut down) of pigment production irrespective of lighting conditions.

The remaining three mutants with insertions in the *mmpL1-crtR* intergenic region displayed YY phenotype (Figure 2a), a property consistent with accumulation of β-carotene irrespective of lighting conditions. The insertions in these mutants were in opposite Tn-*crt* gene orientation and located between the promoter P3 and the transcription start site (TSS2) of a second light-inducible promoter region (P2) driving *crtR* expression (Figure 5 and Figure S2b). We postulate that these insertions created a polar effect lowering *crtR* expression to only that originating from the promoter P3, and that the resulting reduced CrtR levels are not sufficient to repress carotenogenesis genes in the absence of light. Nevertheless, the remaining levels of the repressor are enough to prevent the RR phenotype seen in the *crtR* knockouts (e.g., MK78). Consistent with the proposed rationale behind the YY phenotype of these mutants, doubling the *crtR* gene dosage in MK74 (a *mmpL1-crtR* intergenic region mutant with YY phenotype, Figure 6a) by transformation of the mutant with the P3-*crtR* fragment (which is expected to afford an increase in *crtR* expression level of the order of 2-fold in the mutant) led to full complementation (Figure 6b). Altogether, the results of our analysis of the Tn mutants with insertions in *crtR* and the *mmpL1-crtR* intergenic regions are in line with the proposed role of CrtR as a transcription regulator controlling the photochromogenic phenotype of *Mk*.

**Figure 8:**
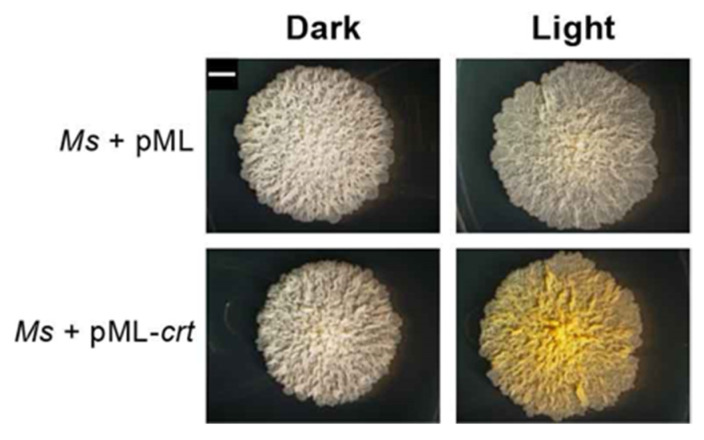
The *M. kansasii* CRT locus confers photochromogenicity to *M. smegmatis*. Representative macrocolonies of *M. smegmatis* (*Ms*) containing the empty pML1335 vector or a vector derivative carrying the CRT locus are shown. Macrocolonies were grown in the dark (‘Dark’ column) or under light exposure (‘Light’ column) for 3 days. White scale bar = 2 mm.

To seek further support for the role of CrtR, we tested whether introduction of the CRT locus fragment into *Mycobacterium smegmatis* (*Ms*) would endow the bacterium with photochromogenicity. Indeed, the *Ms* transformant became clearly photochromogenic. While the parental *Ms* had the same off-white appearance when grown in the dark or under light, the strain carrying the CRT locus retained the WT off-white phenotype in the dark, but developed a light-induced yellow color comparable to that seen in WT *Mk* in response to light exposure (Figure 8). This finding conclusively links the *Mk* CRT locus fragment to photochromogenicity and supports the light-dependent regulatory role of CrtR proposed herein.

Our examination of the correlation between the position and orientation of the insertions upstream of *mmpL1* and the phenotypes of these mutants revealed several interesting patterns as well (Figure 2a). First, the YY phenotype of the *crtE* mutants with Tn-*crtE* co-orientation demonstrated that, unexpectedly, *crtE* is not essential for carotene production. CrtE belongs to the group of geranylgeranyl diphosphate (GGPP) synthases, which are required for synthesis of the carotenoid biosynthetic precursor GGPP from IPP and DMAPP [43]. Thus, our finding indicates the presence of a CrtE-independent route to GGPP in *Mk*. Notably, studies on isoprenoid metabolism in *Mt* have identified alternative *trans*-isoprenyl diphosphate synthases catalyzing GGPP formation [49,50,51]. In view of this precedent, we searched for possible homologues of these *Mt* synthases in *Mk*. The analysis revealed two putative *trans*-isoprenyl diphosphate synthases (i.e., MKAN_RS17995 and MKAN_RS02680) that might provide pathways to GGPP in the *crtE* mutants (Figure 3b). We have determined that, like *crtE*, *MKAN_RS17995* and *MKAN_RS02680* are nonessential [38,52]. The redundancy of genes encoding isoprenyl synthases with GGPP activity would explain the lack of identification of *MKAN_RS17995* and *MKAN_RS02680* Tn mutants in our screen. Furthermore, because the YY phenotype of the *crtE* mutants with Tn-*crtE* co-orientation was independent from light treatment, the expression of any gene required for production of GGPP in these *crtE* knockouts would be expected to take place both in the absence and in the presence of light exposure. This prediction was confirmed for *MKAN_RS17995* and *MKAN_RS02680* by RNA-seq analysis (Figure S2i). In view of our findings, we propose that at least one of the two isoprenyl diphosphate synthase candidates provides GGPP in the absence of CrtE.

**Figure 9:**
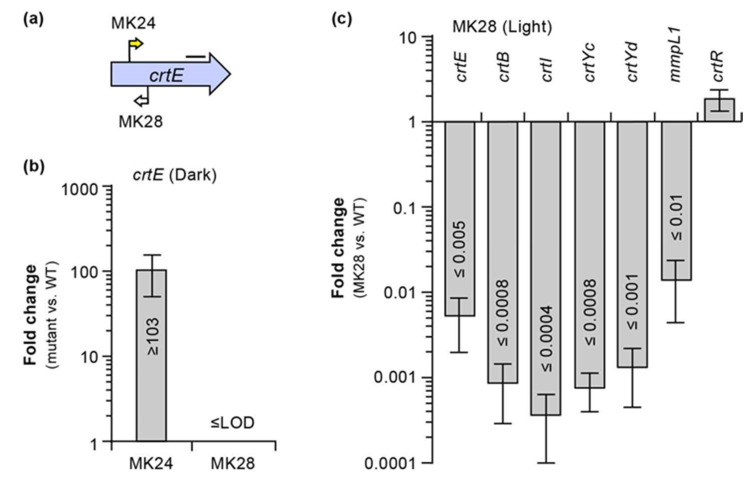
RT-qPCR analysis of the effect of transposon insertion orientation in *crtE* on the expression of CRT locus genes. (**a**) Location of the insertions in MK24 and MK28 and the position of the PCR amplicon of *crtE* (black bar). Arrows demarcating insertions are as noted in Figure 2. (**b**) *crtE* expression levels in MK24 (YY mutant) and MK28 (WW mutant). The value in the bar indicates expression fold change in the mutant relative to the wild-type strain (WT). Cultures were grown in the dark. The ≥ symbol preceding the fold change value denotes that *crtE* expression levels in the WT were at or below the limit of detection (LOD). ≤LOD signifies gene expression levels in both MK28 and WT strains were at or below the LOD. Data represent means ± SEM of two biological replicates. (**c**) Expression levels of *crt* genes and *mmpL1* in MK28 relative to the WT. Cultures were grown in the dark, exposed to light for 20 min, and then subjected to gene expression analysis by strand-specific RT-qPCR. The ≤ symbol preceding a fold change value signifies that gene expression levels in MK28 were at or below the LOD. Data represent means ± SEM of two biological replicates.

Another unexpected finding arising from the analysis of the *crtE* mutants was an unambiguous Tn orientation-phenotype relationship with Tn-*crtE* co-orientation leading to YY mutants and the opposite orientation rendering WW mutants (Figure 2a). This pattern extended to the *crtE*-*crtI* and *MKAN_RS31895-crtE* intergenic regions, with the latter containing a light-inducible promoter region (P1) driving expression of *crt* genes, as determined from our promoter prediction and RNA-seq analyses (Figure 5 and Figure S2a). We hypothesized that the YY phenotype could have arisen from Tn-driven constitutive expression of downstream *crt* genes leading to carotene synthesis in the absence of light. In contrast, we theorized that the WW phenotype could be explained by an insertion-derived polar effect preventing the light-induced expression of *crt* genes located downstream of *crtE* and needed for pigment formation. To probe these ideas, we performed RT-qPCR-based expression analysis using the representative *crtE* mutants MK24 and MK28 (Figure 9a). The data demonstrated Tn-driven overexpression of the *crtE*’s segment downstream of the insertion in the dark for MK24 (YY, Tn-*crtE* co-orientation, Figure 9a) relative to the WT strain corresponding to a >100-fold increase (Figure 9b). Conversely, the same analysis using MK28 (WW, opposite Tn-*crtE* orientation, Figure 9a) revealed no detectable *crtE* expression (Figure 9b). On the other hand, expression analysis of light-exposed cultures of MK28 and the WT strain showed that the Tn caused a polar effect that drastically reduced the transcription of *crt* genes downstream of the Tn insertion (Figure 9c). Consistent with our finding of Tn-driven expression of *crt* genes, we were unable to restore the WT WY phenotype in MK24 by transformation with pML-*crt*, which carried the entire CRT locus fragment (not shown). Moreover, in agreement with the proposed polar effect in MK28, complementation analysis of the mutant revealed no rescue of the WT phenotype by a *crtE* copy under the control of the *crtE* promoter region (P1-*crtE* fragment, pML-*crtE*) (not shown), but the WT phenotype was fully recovered by pML-*crt*, carrying the CRT locus fragment (Figure 6b). The disruption of *crtI*, *crtB*, *crtYc*, *crtYd*, and *mmpL1* expression in MK28 (Figure 9c) supports the idea of these genes being coregulated and in an operon with *crtE*.

A noticeable cluster of insertions with Tn-*crt* co-orientation that correlated with YY phenotype was located at the very 3′ end of *MKAN_RS31895* (Figure 2a), the gene noted above encoding a glycoside hydrolase of unknown function. The phenotype of these mutants indicates that the *MKAN_RS31895* is not essential for pigment production. The lack of *MKAN_RS31895*::Tn isolates with opposite Tn-*crt* gene orientation might suggest that they do not alter the pigmentation phenotype. RNA-seq analysis demonstrated that the gene is not light-regulated along with the *crt* genes (Figure S2c), and our orthology and literature analyses did not uncover any potential link between the gene and carotene metabolism. In agreement with these results, the gene is not present in the *Mk* fragment shown to be sufficient to confer photochromogenicity to *Ms*. Based on these findings, we conclude that the YY phenotype of the *MKAN_RS31895* mutants is not due to a loss of *MKAN_RS31895* function, but it arises from Tn-driven constitutive expression of the downstream *crt* genes.

In contrast to the pattern seen with the *crtE* mutants, insertions in *crtI* or *crtB* led to WW phenotype regardless of Tn orientation (Figure 2a), an outcome in agreement with the expected requirement of the phytoene desaturase (CrtI) and phytoene synthase (CrtB) for carotenoid synthesis (Figure 3a). Furthermore, unlike in the case of *crtE*, no obvious paralogues of *crtI* or *crtB* appear to be present in the genome. The representative *crtI* mutant MK46 and the representative *crtB* mutant MK54 could not be complemented by a WT copy of the disrupted gene under the control of the P1 promoter region (P1-*crtI* and P1-*crtB* fragments; pML-*crtI* and pML-*crtB* plasmids) (not shown), but the mutants regained the WT WY phenotype upon transformation with the entire CRT locus fragment (pML-*crt* plasmid) (Figure 6b). The fact that insertions upstream of *crtI* with Tn-*crt* gene co-orientation led to mutants with YY phenotype (Figure 2a) suggests that the Tn-driven constitutive expression of *crt* genes is sufficient to afford colony pigmentation. Thus, the lack of complementation of MK46 and MK54, two mutants with Tn-*crt* gene co-orientation, by the single genes (P1-*crtI* and P1-*crtB* fragments) is unlikely to be caused by a loss of transcription due to a polar effect on genes downstream of the insertions. A plausible explanation for the lack of complementation with single genes is a drastic reduction in Crt protein synthesis in these mutants arising from loss of critical translational coupling between overlapping contiguous open reading frames (e.g., *crtI-crtB* and *crtB-crtYc*) (Figure S5). The translational coupling might be needed for efficient translation, correct protein complex formation, and/or protein (co-)folding [53]. In fact, several lines of evidence suggest that the enzymes phytoene desaturase, phytoene synthase, and lycopene cyclase operate as a complex assembled at the membrane and involved in substrate channeling [54,55]. Thus, it is reasonable to speculate that translational coupling might be critical for a proper quaternary structure assembly of the *Mk* enzyme complex.

Interestingly, insertions disrupting *crtYc* or *crtYd* (predicted heterodimeric lycopene β-cyclase) rendered mutants with WO phenotype (Figure 2a). The representative *crtYc* mutant MK71 and the representative *crtYd* mutant MK73 (Figure 6a) appeared to be similarly complemented by a WT copy of the corresponding disrupted gene expressed from the P1 promoter region, or by the CRT locus fragment (Figure 6b). In all cases, complementation produced the shift of the light-induced orange tint of the mutants towards a more yellow coloration comparable to that in the WT strain. Heterodimeric lycopene cyclases carry out the cyclization of both ends of lycopene to produce β-carotene (Figure 3a). The orange tint of the *crtYc* and *crtYd* mutants exposed to light appeared to be an intermediate between the red of lycopene and the yellow of β-carotene. Notably, monocyclic γ-carotene, which has been detected in *Mk* [41], exhibits an absorption spectrum between those of lycopene and β-carotene and is red-orange [56]. Thus, our results might suggest that the insertions in *crtYc* or *crtYd* led to loss of both CrtYc-CrtYd heterodimer formation and bi-cyclization activity, but retention of mono-cyclization activity and consequent accumulation of γ-carotene upon light exposure. However, the possibility that the orange tint seen in the mutants results from accumulation of some red lycopene along with some of γ-carotene and/or β-carotene (indicating retention of some level of bi-cyclization activity) cannot be ruled out. Future studies to investigate the types and relative quantities of the carotenoids accumulated in these and other pigmentation mutants identified herein will be warranted.

Unexpectedly, all 19 insertions identified in the *crtYc-crtYd* segment had opposite Tn-*crt* gene orientation. This puzzling insertion pattern is unlikely due to chance, and it might suggest that Tn-gene co-orientation, for unclear reasons, does not lead to a detectable pigmentation anomaly. Alternatively, Tn-driven constitutive overexpression of *mmpL1* in *crtYc-crtYd* mutants with Tn-*crt* gene co-orientation might compromise viability, thus eliminating these mutants from our library.

### 2.4. Bioinformatic and Insertional Analysis of the Carotenoid Cleavage Oxygenase Locus

We identified 24 insertions mapping to a locus (i.e., the CCO locus) containing two divergently transcribed genes of unknown function (Figure 2b). Twenty of these insertions mapped to a gene encoding a predicted TetR/AcrR family regulator (*MKAN_RS18575*, hereafter referred to as *ccoR^Mk^*) and correlated with WW phenotype. The remaining insertions mapped to *MKAN_RS18580* (hereafter referred to as *cco1^Mk^*), and led to YY phenotype (Figure 2b). The gene *cco1^Mk^* is one of two predicted paralogues encoding carotenoid oxygenase family proteins of unknown function in *Mk*, with the second being *MKAN_RS08870*.

**Figure 10:**
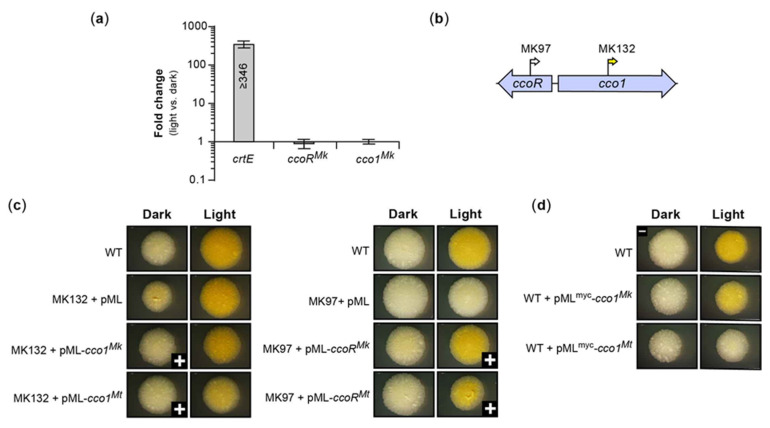
Influence of light on the expression of CCO locus genes, phenotype of representative CCO locus mutants, and effect of *cco1* overexpression. (**a**) RT-qPCR analysis of the effect of light exposure on the expression of *cco1^Mk^* and *ccoR^Mk^* in *M. kansasii* wild-type (WT). The values in the bars indicate expression fold changes in light-treated cultures relative to untreated cultures. Data represent means ± SEM of three biological replicates. The ≥ symbol preceding the fold change value denotes that *crtE* expression levels in the dark condition were at or below the limit of detection (LOD). (**b**) CCO locus diagram with arrows demarcating the insertions in the representative mutants MK97 (WW phenotype) and MK132 (YY phenotype). (**c**) Macrocolony phenotype. The mutants and the WT strain (WY phenotype) shown in both ‘Dark’ and ‘Light’ columns were grown on two replicate plates in the dark (~11 d). Then, one replicate was kept in the dark (‘Dark’ columns) and the other was exposed to light (~3 h, ‘Light’ columns). Both replicates were subsequently incubated for growth in the dark (3 d) and imaged. pML, empty pML1335 vector. The complementation control strains carried the indicated pML1335 derivatives expressing specific mycobacterial genes (Table S2). The + symbol in the lower right corner of the images indicates full complementation (i.e., WY phenotype). (**d**) Constitutive overexpression of *cco1^Mk^* or *cco1^Mt^* in the WT strain. White scale bar (top left image) = 2 mm. The results shown are representative of three independent experiments.

Despite the involvement of the CCO locus in carotenoid metabolism revealed by the phenotype of its mutants, the expression of *cco1^Mk^* and *ccoR^Mk^* was not influenced by light. RT-qPCR analysis comparing *cco* gene expression in cultures with and without light exposure demonstrated that neither of the genes were differentially expressed in response to light (Figure 10a), a finding supported by RNA-Seq analysis (Figure S2d). In contrast, the expression analysis of *crtE* (included in the experiments as a light-inducible gene control) showed the expected drastic upregulation in response to light treatment.

Notably, as shown for the representative mutant MK132 (Figure 10b), the yellow pigmentation shown by the *cco1^Mk^* mutant in the dark was considerably less intense than the yellow color induced in the mutant by light exposure (Figure 10c). However, the latter pigmentation was comparable to that developed in the WT exposed to light (Figure 10c). Interestingly, the non-carotenoid producer *Mt* has an orthologous regulator-carotenoid cleavage oxygenase locus, *Rv0653c*-*Rv0654* (hereafter referred to as *ccoR^Mt^*-*cco1^Mt^*), and the Rv0654 enzyme (hereafter referred to as Cco1^Mt^, 75% amino acid identity with Cco1^Mk^) has been shown to cleave several carotenoids and apocarotenoids, including β-carotene [57].

Complementation analysis of the representative *cco1^Mk^* mutant MK132 (Figure 10b, YY phenotype) showed restoration of the WT phenotype upon transformation with a *cco1^Mk^* copy expressed from its predicted promoter region (i.e., *ccoR^Mk^*-*cco1^Mk^* intergenic region) (Figure 10c). Notably, MK132 was also complemented by the orthologous DNA segment of *Mt* (promoter-*cco1^Mt^*) from the *ccoR^Mt^*-*cco1^Mt^* locus (Figure 10c), thus demonstrating functional equivalence. Interestingly, overexpression of either of the *cco1* orthologues under the control of the heterologous strong constitutive Pmyc1 promoter in the WT strain led to a modest reduction in the intensity (for *cco1^Mk^*) or a complete disappearance (for *cco1^Mt^*) of the yellow coloration characteristic of the light-exposed WT strain (Figure 10d). The finding could perhaps be explained by carotenoid cleavage oxygenase overexpression-dependent degradation of cellular carotenes.

**Figure 11:**
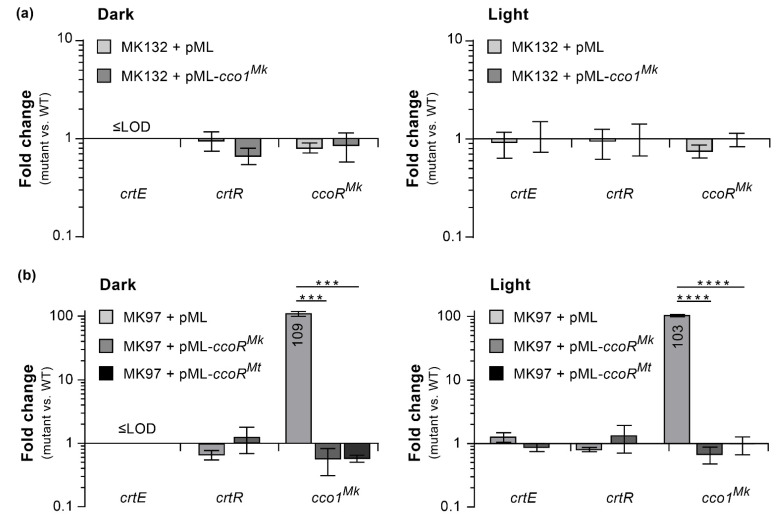
RT-qPCR analysis of the effect of the disruption of *cco1^Mk^* or *ccoR^Mk^* on the expression of genes in the CCO and CRT loci. (**a**) Impact of the *cco1^Mk^* disruption in MK132 (YY mutant). (**b**) Impact of the *ccoR^Mk^* disruption in MK97 (WW mutant). The values in the bars indicate expression fold changes in the mutant relative to the wild-type (WT). All cultures were grown in the dark and then split into two. One of the two cultures was subjected to a 20 min light treatment (graphs on the right), while the other was kept shielded from light (graphs on the left). ≤LOD indicates that the expression values in both mutant and WT strains were at or below the limit of detection (LOD). In (**b**) statistical significance was determined using an unpaired, two-tailed *t*-test with the Holm–Sidak method in GraphPad PRISM 6 (***, *p* < 0.0005; ****, *p* < 0.00005). Strand specific RT-qPCR was used for the determinations. Data represent means ± SEM of three biological replicates, except for the dark condition graph in (**a**), which is derived from two replicates.

Lastly, we carried out RT-qPCR analysis to assess whether the loss of *cco1^Mk^* in MK132 had impacted expression of its *ccoR^Mk^* neighbor, the light-induced *crtE* (as representative of the *crtEIBYcYd* gene cluster), or *crtR*. The results indicated that loss of *cco1^Mk^* had no appreciable effect on the expression of these genes, irrespective of light conditions (Figure 11a). Altogether, these findings lead us to propose that the slight yellow pigmentation seen in the *cco1^Mk^* mutants in the absence of light exposure indicates that Cco1^Mk^ might be involved in cleavage of trace amounts of carotene produced in the dark due to incomplete pathway repression leading to occasional transcription escape events in the absence of light. Such a function could be part of a degradation pathway for recycling carotenes into raw materials for other cellular processes and/or generating apocarotenoids with physiological functions yet unknown.

Complementation analysis of the representative *ccoR^Mk^* mutant MK97 (Figure 10b; WW phenotype) revealed recovery of the WT WY phenotype by a *ccoR^Mk^* copy expressed from its predicted promoter region (i.e., *ccoR^Mk^*-*cco1^Mk^* intergenic region) (Figure 10c). Paralleling the complementation result seen with the *cco1^Mk^* mutant, MK97 was also complemented by the orthologous DNA segment of *Mt* from the *ccoR^Mt^*-*cco1^Mt^* locus (Figure 10c). The WW phenotype of the *ccoR^Mk^* mutants indicated that they failed to develop pigmentation regardless of lighting conditions, thus revealing a central role for the regulator in pigment metabolism. We reasoned that the absence of light-inducible pigmentation of the *ccoR^Mk^* mutants could perhaps arise from lack of expression of carotenoid biosynthetic genes. This scenario could be explained if CcoR^Mk^ were to function as a critical activator of carotene biosynthetic gene transcription or as a repressor of *crtR* transcription, two options that are not mutually exclusive. Alternatively, however, the phenotype of the *ccoR^Mk^* mutants could be produced by a drastic upregulation of Cco1^Mk^ expression leading to a depletion of carotenes, a view consistent with the phenotypic outcome noted above for the constitutive Cco1^Mk^ and Cco1^Mt^ overexpression in the WT strain (Figure 10d). To investigate these possibilities, we evaluated the effect of *ccoR^Mk^* disruption on the expression of *cco1^Mk^*, *crtE* (as representative of the light-induced *crtEIBYcYd* gene cluster), and *crtR* by RT-qPCR analysis. The results revealed that loss of *ccoR^Mk^* in the representative MK97 mutant led to no expression changes of *crtE* or *crtR*, but produced a drastic 100-fold upregulation of *cco1^Mk^* relative to WT irrespective of lighting conditions (Figure 11b). Conversely, the *ccoR^Mk^*- and *ccoR^Mt^*-complemented mutant displayed expression patterns indistinguishable from those of the WT strain (Figure 11b). Thus, the RT-qPCR analysis is consistent with the idea that CcoR^Mk^ functions as a repressor of *cco1^Mk^* expression irrespective of lighting conditions. It is likely that the same regulatory relationship exists between *ccoR^Mt^* and *cco1^Mt^*. In view of our findings, we postulate that the absence of light-induced pigmentation in the *ccoR^Mk^* mutants results from an exacerbated Cco1^Mk^-dependent carotene degradation, secondary to *cco1^Mk^* overexpression. Our findings highlight CcoR^Mk^ as a gatekeeper of a Cco1^Mk^-dependent carotenoid breakdown pathway leading to production of apocarotenoid compounds. The conditions that determine the physiological modulation of such a pathway remain to be elucidated.

### 2.5. Bioinformatic and Insertional Analysis of the fnr1-desA3 Locus

**Figure 12:**
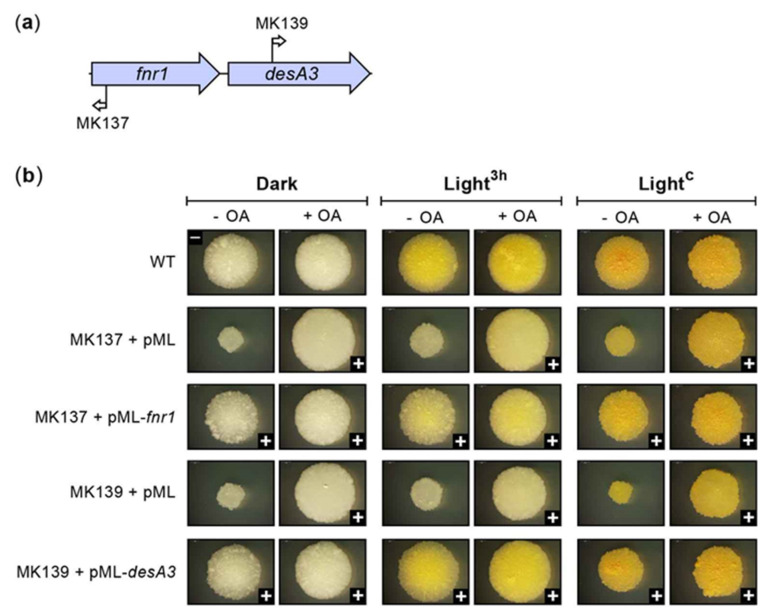
Macrocolony phenotype of representative FD locus mutants and their respective complementation control strains. (**a**) FD locus diagram with arrows demarcating the insertions in MK137 and MK139 (WW mutants). (**b**) Macrocolony phenotype. The mutant strains and the wild-type (WT) reference (WY phenotype) were grown in the dark for 13 days (‘Dark’ column), in the dark for 10 days, then exposed to light for 3 h and incubated in the dark for an additional three days (‘Light^3h^’ column), or under continuous light exposure for 13 days (‘Light^c^’ column). +/- OA, medium with/without oleic acid (50 mg/L). pML, empty pML1335 vector. The complementation control strains carried the indicated pML1335 derivatives expressing specific mycobacterial genes (Table S2). A + symbol in the lower right corner of the image indicates nearly full to full complementation. White scale bar (top left image) = 2 mm. Results shown are representative of three experiments.

We identified five insertions leading to WW phenotype and reduced colony growth rate that mapped to a two-gene locus (i.e., FD locus) located in a chromosomal region distant from the CRT and CCO loci noted above (Figure 2c). Two of the insertions were in *MKAN_RS20530* (hereafter referred to as *fnr1*), a gene encoding a putative ferredoxin-NADP(H) reductase (FNR) of unknown function. The remaining three insertions mapped to *MKAN_RS20535* (hereafter referred to as *desA3*), which encodes a predicted fatty acid desaturase of unknown function. Unlike the CRT locus genes, the expression of *fnr1* and *desA3* was not influenced by light exposure (Figure S2e). Complementation analysis of the representative *fnr1* and *desA3* mutants MK137 and MK139 (Figure 12a), respectively, showed complete (or nearly complete) recovery of WT growth and pigmentation upon transformation with a WT copy of the disrupted gene expressed from its predicted promoter region (Figure 12b). These findings link the loss of gene function and the mutant phenotype. The gene *fnr1* has four paralogues (*MKAN_RS01630*, *MKAN_RS11415*, *MKAN_RS11565*, and *MKAN_RS15180*), whereas *desA3* has two (*MKAN_RS20540*, located adjacent to *desA3*, and *MKAN_RS15185*). Interestingly, the *fnr1* paralogue *MKAN_RS15180* pairs with the *desA3* paralogue *MKAN_RS15185* in an organization equivalent to that seen for *fnr1-desA3.* None of the *fnr1* or *desA3* paralogues are essential [38,52] and, based on their number of TA sites, the probability of the Tn missing any of these genes by chance is ≤10^−8^ (Figure S1). Thus, we speculate that it is unlikely that insertions in any of these paralogues lead to a noticeable pigmentation phenotype.

Notably, our orthology analysis revealed that the *Mk fnr1-desA3* pair is orthologous to the non-essential *Rv3230c-Rv3229c* and *MSMEG_1885*-*MSMEG_1886* pairs of *Mt* and *Ms*, respectively. Several studies indicated that the latter pair encodes a ferredoxin-NADP(H) reductase and a membrane-bound NADPH-dependent stearoyl-CoA 9-desaturase that partner to synthesize oleic acid (OA) [58,59,60,61,62], a critical cell membrane component. Thus, the orthology analysis points to a function of *Mk fnr1-desA3* in OA synthesis. In connection with this idea, it is noteworthy that the *Mk fnr1* and *desA3* mutants showed a marked growth defect (Figure 12b), a phenotype that would be consistent with an OA limitation. Interestingly, the growth defect of these mutants mirrored that seen in the *Ms MSMEG_1886* (*desA3* orthologue) knockout, a mutant phenotype remediated by exogenous OA [58]. These observations led us to assess whether our *fnr1* and *desA3* mutants would recover the WT phenotype when grown on agar plates supplemented with OA. The results of this analysis revealed that, as expected, OA had no impact on the growth or photochromogenicity of the WT strain control or the phenotype of the carotene biosynthesis deficient *crtE* mutant (MK28, WW phenotype control; not shown). However, OA had a clear effect on the FD locus mutants. It led to restoration of WT growth, essentially phenocopying the genetic complementation (Figure 12b). The supplementation also partially corrected the pigment photoinduction defect of the mutants seen in response to the standard 3 h light treatment (Figure 12b). The light treatment induced a very faint yellow pigmentation in the mutants that was slightly more intense on the OA-containing plates than in the OA-free plates (Figure 12b). Interestingly, the *fnr1* and *desA3* mutants grown under constant illumination developed the characteristic pigmentation phenotype we established for the WT strain under this lighting condition (i.e., intense yellow with red specks, presumably enriched in lycopene; Figure S4) irrespective of OA supplementation status (Figure 12b). As anticipated, however, the control *crtE* mutant (MK28) remained unpigmented despite constant illumination (not shown). In all, our findings support the proposed role of the FD locus in OA synthesis and the view that OA limitation is responsible for the growth defect of the *fnr1* and *desA3* mutants. Moreover, the findings also indicate that neither *fnr1* nor *desA3* is essential for carotene production and suggest that pigment accumulation in the mutants proceeds with a lower efficiency than that of the WT strain. Lastly, the results also decoupled the growth and pigmentation defects of the FD locus mutants, i.e., WT-like growth is not necessary for photoinduction of pigment production, and vice versa.

As noted above, our data suggest a less efficient pigment accumulation in the FD locus mutants than in the WT strain. A possible basis for this phenotype could be an impairment of protein-membrane interaction dependent functions essential for carotenogenesis, owing to abnormalities in membrane properties in the FD locus mutants. Such abnormalities could be caused by the OA limitation in the mutants. This speculation is supported by several lines of evidence suggesting that the enzymes phytoene synthase, phytoene desaturase, and lycopene cyclase operate as a complex assembled at the membrane and involved in substrate channeling [54,55]. Thus, it is tempting to speculate that abnormal membrane properties in the mutant hindered optimal formation of a properly functioning CrtBIYcYd complex critical for carotenogenesis in *Mk*.

### 2.6. Transcriptome Changes Induced by Light in M. kansasii

To gain a first insight into the genome-wide transcriptional response of *Mk* to light and provide context for and inform the interpretation of our insertional analysis, we compared RNA-seq expression profiles of *Mk* cultures with or without light exposure (20 min). The analysis revealed only 14 differentially expressed genes. These genes were all upregulated in response to light and distributed across four distinct chromosomal loci (Table 1 and Figure S6). In agreement with our RT-qPCR analysis of the CRT locus, 8 of the 14 differentially expressed genes were the light-inducible *crt*, *mmpL1*, and *fni* genes (Figure S2a,b). Notably, *crtR* is one of the genes upregulated in response to light treatment. It is tempting to speculate that this *crtR* upregulation represents a feedback loop evolved to prevent unnecessary or disadvantageous pigment overproduction by providing an increase in CrtR-dependent pathway repression power after a light-induced pathway upregulation and consequent pigment biosynthesis burst.

The second locus (hereafter referred to as the photolyase locus) with differentially expressed genes included a three-gene cluster predicted to encode a deoxyribodipyrimidine photolyase (MKAN_RS22245), a tryptophan-rich sensory protein/translocator protein family member (MKAN_RS22250), and an MmpL family transporter (MKAN_RS22255) (hereafter referred to as *phrB*, *tspO*, and *mmpL2*, respectively) (Figure S2f). Interestingly, photolyases are critical for repairing light-induced DNA lesions, and light-dependent photolyase upregulation was recently reported in *Streptomyces lividans* [63]. TspO proteins are transmembrane, conserved throughout evolution, and involved in a myriad of cell processes through their ability to bind tetrapyrroles such as porphyrins [64,65]. Notably, expression of a *tspO* gene in the cyanobacterium *Fremyella diplosiphon* was found to be upregulated in response to light, nutrient deficiency, and salt, osmotic, or oxidative stress [66]. Lastly, a TspO protein of the photosynthetic purple bacterium *Rhodobacter sphaeroides* has been postulated to be involved in sensing oxygen and perhaps light, and the loss of the *tspO* gene rendered a mutant with increased carotenoid pigment production [67,68].

The third light-induced locus included *MKAN_RS11600* and *MKAN_RS11610* (hereafter referred to as *mpk83* and *mpk70*, respectively) (Figure S2g), two conserved genes encoding the homologues of the precursors for the mycobacterial antigenic proteins MPT83/MPB83 and MPT70/MPB70 (*Mt*/*Mycobacterium bovis* BCG designations), respectively [69,70]. The genes *mpk83* and *mpk70*, along with *dipZ* (*MKAN_RS11605*) located between them, correspond to a gene triad conserved in *Mt* and other slow-growing pathogenic mycobacteria [71]. Notably, despite the position of *dipZ* in *Mk*, its expression was not differentially regulated by light exposure. The conserved mycobacterial *dipZ* gene encodes a possible C-type cytochrome biogenesis protein of the CcdA-family and is potentially involved in formation of internal disulfide bridges of MPT83/MPB83 and MPT70/MPB70 [69,72,73]. Interestingly, the *Mt mpt83, mpt70,* and *dipZ* genes have each a SigK-dependent promoter and are regulated by the sigma factor–anti-sigma factor system SigK-RskA in response to environmental stimuli that remain obscure [70,71,74]. While there are no reported data regarding the regulation of *Mk mpk83, mpk70*, and *dipZ*, there is a putative SigK binding site upstream of each of these genes [71], suggesting that they might be regulated by the *Mk* SigK-RskA system (i.e., MKAN_RS17285-MKAN_RS17280, as per our orthology analysis). To our knowledge, the findings reported herein provide the first demonstration of light-dependent regulation of mycobacterial antigenic proteins. Lastly, the fourth upregulated locus contains the gene *MKAN_RS19770*, encoding a member of the mycobacterial PE protein family of unknown function (Figure S2h). It is worth noting that the magnitude of the light-induced upregulation of *mpk83, mpk70*, and *MKAN_RS19770* was considerably smaller than that seen for genes in the CRT and photolyase loci. To our knowledge, there are no reports connecting mycobacterial *mpk83, mpk70*, or *MKAN_RS19770* orthologues with light-regulated processes or carotenoid pigment production. The physiological advantage of having these genes upregulated by light exposure remains unclear.

Of note, none of the genes in the last three loci noted above are essential [38,52], and thus lack of Tn mutant viability is unlikely to have excluded their mutants from our Tn library. We speculate that the lack of Tn mutants of these genes among the isolates analyzed is unlikely due to chance (Figure S1), and suggests that insertions in any of these genes do not have a conspicuous impact on the pigmentation phenotype of the mutants.

Overall, the short 20 min-light exposure elicited a focused transcriptome response consisting of only 14 differentially expressed genes, a number representing ~0.25% of the genes present in *Mk*. Moreover, 9 of the upregulated genes can be hypothesized to be linked to physiological processes influenced by or related to light exposure. Thus, we conclude that our short light treatment led to differentially expressed gene information unlikely to be significantly confounded by indirect gene expression fluctuations secondary to changes in cellular physiology brought about by the transcriptional response directly triggered by light. Moreover, in agreement with the observation that carotenoid accumulation in *Mk* is not detected during the first 60-90 min of light exposure [75], the 20 min light exposure used in our studies was not sufficient to produce visible accumulation of pigments in *Mk* cultures or colonies (without subsequent incubation for growth; not shown). Thus, the changes in gene expression we observed are unlikely to be confounded by a physiologic response caused by the accumulation of pigments in the cell. To our knowledge, our results provide the first insight into the genome-wide transcriptional response to light in mycobacteria.

## 3. Materials and Methods

### 3.1. Routine Culturing Conditions, Molecular Biology Techniques, and Reagents

Unless otherwise stated, *Mk* (reference strain ATCC 12478; Hauduroy), *Mycobacterium smegmatis* (*Ms*; strain mc^2^155, ATCC 700084), and their derivatives were cultured under standard conditions in Middlebrook 7H9 broth (Difco, Becton-Dickinson and Co., Franklin Lakes, NJ, United States) supplemented with 10% ADN (5% bovine serum albumin [BSA], 2% dextrose, 0.85% NaCl) and 0.05% Tween 80 (s7H9 broth), or ADN-supplemented Middlebrook 7H11 (s7H11) or 7H10 (s7H10) agar (Difco) as reported [38]. Unless otherwise indicated, cultures were grown in the dark and shielded from white (ambient) light exposure during their manipulations, which were carried out under red light illumination (25 W red incandescent light) as needed. Where appropriate, kanamycin (Km, 30 μg/mL) and/or hygromycin (Hyg, 150 μg/mL) were added to the Middlebrook media. Culturing of *Escherichia coli* strains was carried out under standard conditions in Luria–Bertani media [76]. When required, ampicillin (Amp, 100 μg/mL), Km (30–50 μg/mL), Hyg (200 μg/mL), and/or 5-bromo-4-chloro-3-indolyl-β-d-galactopyranoside (X-Gal, 20-30 μg/mL) were added to the Luria–Bertani media. Routine DNA manipulations were carried out using established protocols and, unless otherwise noted, using *E. coli* DH5α as the primary cloning host [76]. PCR-generated DNA fragments used in plasmid constructions were sequenced to verify fidelity (Genewiz, Inc., Chelmsford, MA, United States). Plasmid electroporation into mycobacteria and selection of mycobacterial transformants were performed following standard methodologies [38]. All mycobacterial strains were subjected to two rounds of strain purification by streaking for colony isolation. Images of mycobacterial colonies and macrocolonies (arising from spot-inoculation of liquid cultures onto agar plates [38,77]) were digitally captured using an Olympus SZX7 stereo microscope (Olympus Life Science, Waltham, MA, United States) or a DSLR camera (Canon Inc., Melville, NY, United States). Unless otherwise noted, reagents were purchased from Thermo Fisher Scientific Inc. (Branchburg, NJ, United States), New England Biolabs Inc. (Ipswich, MA, United States), VWR International LLC (Radnor, PA, United States), Sigma-Aldrich Inc. (St. Louis, MO, United States), or Qiagen LLC (Germantown, MD, United States).

### 3.2. Preparation of the M. kansasii Transposon Library and Screening for Pigmentation Mutants

High-titer stocks of phage ϕMycoMarT7 were generated and titrated using *Ms* as reported [42]. The stocks were used to transduce *Mk* for generation of libraries of Tn mutants as we recently described [38]. After transduction, the transduction mixtures were pelleted, re-suspended in growth medium with 25% glycerol, aliquoted, and stored at −80 °C until needed. Aliquots of the transduction mixtures were plated out for enumeration of the number of Km resistant (Km^R^) CFU/mL, a proxy for the number of Tn mutants/mL. The titrated library aliquots were plated out on s7H11 agar (15 cm diameter plates) with Km, and the inoculated plates were incubated for colony development (37 °C, ~2 wks, in the dark). After incubation, images of the screen plates were digitally captured with a DSLR camera and the plates were subsequently screened for mutants with pigmentation phenotypes differing from the unpigmented (off-white) wild-type phenotype of the majority of colonies on the screen plates. Screening was done by naked eye (with occasional assistance of a magnifying lens) under ambient (white, fluorescent) light. The pigmentation mutants identified (mutant pool 1) on the screen plates were picked and streaked for colony isolation on Km-containing s7H11 plates that were then incubated for colony development (in the dark, 37 °C, 2–3 wks). Immediately after pigmentation mutant picking, the screen plates were exposed to fluorescent light for 2–3 h on a benchtop and returned to incubation (in the dark, 37 °C, 2–3 d) to allow for expression of light-induced pigmentation. After this incubation, the screen plates were again imaged with a DSLR camera and subsequently examined for mutants differing in phenotype from the yellow (wild-type) color of the majority of the colonies on the screen plates. All pigmentation mutants identified (mutant pool 2) on these plates were picked and subjected to colony isolation as noted above for mutant pool 1 isolates. All isolates in the mutant pools 1 and 2 were subjected to confirmation of their pigmentation phenotypes with and without light treatment (~3 h). All mutant isolates were then cultured (in the dark) to saturation in Km-containing s7H9 broth, the bacteria were harvested and resuspended in sterile s7H9 with 25% glycerol, and the suspensions were stored at −80 °C until needed.

### 3.3. Insertion Site Determination and Southern Blot Hybridization Analysis

Insertion sites were identified by sequencing (Genewiz, Inc.) Tn-genome junctions obtained by the plasmid rescue method as we recently reported [38], the arbitrarily primed PCR (AP-PCR) method [78], or by locus-targeted PCR with target locus-specific primer pairs. The latter method was used when the phenotype of the mutant suggested the location of its insertion based on information gathered from already characterized mutants. Genomic DNA needed for the plasmid rescue method was isolated following standard protocols [79]. Genomic DNA used as PCR template was routinely purified using an in-house protocol (hereafter referred to as HAGRID; see Supplementary Materials), which was developed as a variation of published protocols [79,80]. The genomic sequences identified were mapped to the *Mk* genome (NCBI reference sequences NC_022663.1 and NC_022654.1) using nucleotide BLAST (https://blast.ncbi.nlm.nih.gov/BLAST, accessed on 18 November 2022) to determine the insertion site. Southern blot hybridization analysis for verification of Tn insertions in selected strains (Figure S7) was performed on genomic DNA (isolated with the HAGRID method) digested with AatII, and using a Tn-specific 512-bp DIG-labeled DNA hybridization probe as reported [38].

### 3.4. Construction of Plasmids

Plasmid pCP0h was generated by inserting the Hyg resistance cassette (HpaI-PmeI fragment) from plasmid pML1335 [81] into the DraI restriction site of pCP0 [82]. To obtain plasmid pML-*crt,* a PCR-generated fragment (primers NJ1 and NJ2) containing *crtEIBYcYd-mmpL1-crtR* (*MKAN_RS10020 – MKAN_RS10055*; chromosome coordinates: 2342585 to 2350376) was cloned into pCR2.1-TOPO (TOPO TA cloning kit, Invitrogen, Waltham, MA, United States). Subsequently, the insert in the pCR-2.1-TOPO construct was recovered as a PsiI-MfeI fragment and cloned into a pMLΔxylE vector backbone obtained by digestion of pML1335-WCB3 [83] with PsiI and MfeI. The cloned insert included the native CRT locus’ promoter region upstream of *crtE* (P1). To create pML-*cco1^Mk-part^-ccoR^Mk^*, a PCR-generated fragment (primers NJ46 and NJ47) containing a part of the *Mk cco1-ccoR* locus (partial *MKAN_RS18580* and entire *MKAN_RS18575*; genomic coordinates 4262321 to 4263914) was digested with BspHI and MfeI, and then cloned into a pMLΔxylE backbone obtained by digestion of pML-*crt* with BspHI and MfeI. To construct pML-*crtE*, a PCR-generated fragment (chromosome coordinates 2342585 to 2344128; primers NJ112 and NJ101) containing the CRT locus’ promoter P1 region and *crtE* (*MKAN_RS10020)* was digested with AflII and MfeI, and then cloned into a pMLΔxylE backbone obtained by digestion of pML-*ccoR^Mk^* with AflII and MfeI. The plasmid expressed *crtE* from its native promoter region (P1). To create pML-*crtI*, a PCR-generated fragment (primers NJ112 and NJ102) containing the CRT locus’ promoter P1 region (chromosome coordinates 2342585 to 2343093) and a PCR-generated fragment (primers NJ103 and NJ104) containing *crtI* (*MKAN_RS10025*, chromosome coordinates 2344145 to 2345683) were combined using overlap-extension PCR (primers NJ112 and NJ104). The resulting fragment was digested with AflII and MfeI, and then cloned into a pMLΔxylE backbone obtained by digestion of pML-*ccoR^Mk^* with AflII and MfeI. The construction placed *crtI* under the control of promoter P1 region. To obtain pML-*crtB*, a PCR-generated fragment (primers NJ112 and NJ102) containing the CRT locus’ promoter P1 region (chromosome coordinates 2342585 to 2343093) and a PCR-generated fragment (primers NJ105 and NJ106) containing *crtB* (*MKAN_RS10030*, chromosome coordinates 2345683 to 2346642) were combined using overlap-extension PCR (primers NJ112 and NJ106). The resulting fragment was digested with AflII and MfeI, and then cloned into a pMLΔxylE backbone obtained by digestion of pML-*ccoR^Mk^* with AflII and MfeI. The construction placed *crtB* under the control of promoter P1 region. To construct pML-*crtYc*, a PCR-generated fragment (primers NJ112 and NJ102) containing the CRT locus’ promoter P1 region (chromosome coordinates 2342585 to 2343093) and a PCR-generated fragment (primers NJ107 and NJ108) containing *crtYc* (*MKAN_RS10035*, chromosome coordinates 2346642 to 2346963) were combined using overlap-extension PCR (primers NJ112 and NJ108). The resulting fragment was digested with AflII and MfeI, and then cloned into a pMLΔxylE backbone obtained by digestion of pML-*ccoR^Mk^* with AflII and MfeI. The construction placed *crtYc* under the control of promoter P1. To obtain pML-*crtYd*, a PCR-generated fragment (primers NJ112 and NJ102) containing the CRT locus’ promoter P1 and a PCR-generated fragment (primers NJ109 and NJ110) containing *crtYd* (*MKAN_RS10040*; chromosome coordinates 2346962 to 2347270) were combined using overlap-extension PCR (primers NJ112 and NJ110). The resulting fragment was digested with AflII and MfeI, and then cloned into a pMLΔxylE backbone obtained by digestion of pML-*ccoR^Mk^* with AflII and MfeI. The construction placed *crtYd* under the control of promoter P1. To obtain pML^myc^-*crtYd*, a PCR-generated fragment (primers NJ147 and NJ145) containing *crtYd* (*MKAN_RS10040*; chromosome coordinates 2346962 to 2347270) was digested with AflII and SbfI, and then cloned into a pMLΔxylE backbone obtained by digestion of pML^myc^-*cco1^Mk^* (see below) with AflII and SbfI. The construction placed *crtYd* under the control of the mycobacterial optimized promoter *Pmyc1* [84]. To generate pML-*crtR*, a PCR-generated fragment (chromosome coordinates 2342585 to 2349511; primers NJ3 and NJ2) containing *crtR* (*MKAN_RS10050*) and its native promoter region (P3) was cloned into pCR2.1-TOPO. Then, the insert in the pCR-2.1-TOPO construct was recovered as a PsiI-MfeI excerpt and cloned into a pMLΔxylE backbone obtained by digestion of pML1335-WCB3 [83] with PsiI and MfeI. The plasmid expressed *crtR* from its native promoter region. To build pML-*ccoR^Mk^*, a PCR-generated fragment (chromosome coordinates 4262322 to 4263111; primers NJ56 and NJ57) containing *ccoR^Mk^* (*MKAN_RS18575*) and its promoter region was digested with AflII and MfeI, and then cloned into a pMLΔxylE backbone obtained by digestion of pML-*cco1^Mk-part^-ccoR^Mk^* with AflII and MfeI. The plasmid expressed *ccoR^Mk^* from its native promoter. To obtain pML-*cco1^Mk^*, a PCR-generated fragment (chromosome coordinates 4262909 to 4264616; primers NJ111 and NJ78) containing *cco1^Mk^* (*MKAN_RS18580*) and its promoter region was digested with AflII and MfeI, and then cloned into a pMLΔxylE backbone obtained by digestion of plasmid pML-*ccoR^Mk^* with AflII and MfeI. The plasmid expressed *cco1^Mk^* from its native promoter. To construct pML^myc^-*cco1^Mk^*, a PCR-generated fragment (primers NJ64 and NJ65) containing *cco1^Mk^* (*MKAN_RS18580*; chromosome coordinates 4263096 to 4264616) was digested with AflII and SbfI, and then cloned into a pMLΔxylE backbone obtained by AflII-SbfI digestion of pML1335-Pmyc1tetO-*eccB3* [85]. The construction placed *cco1^Mk^* under the control of the mycobacterial optimized promoter *Pmyc1* [84]. To obtain pML-*cco1^Mt^*, a PCR-generated fragment (*Mt* chromosome coordinates 749930 to 751505; GenBank: NC_000962.3; primers NJ79 and NJ80) containing *cco1^Mt^* (*Rv0654*) and its promoter region was digested with AflII and MfeI, and then cloned into a pMLΔxylE backbone obtained by digestion of pML-*ccoR^Mk^* with AflII and MfeI. The plasmid expressed *cco1^Mt^* from its native promoter. To create pML-*ccoR^Mt^*, a PCR-generated fragment (*Mt* chromosome coordinates: 749235 and 750020; primers NJ58 and NJ59) containing *ccoR^Mt^* (*Rv0653c*) and its promoter region was digested with AflII and MfeI, and then cloned into a pMLΔxylE backbone obtained by digestion of pML-*cco1^Mk-part^-ccoR^Mk^* with AflII and MfeI. The plasmid expressed *ccoR^Mt^* from its native promoter. To build pML^myc^-*cco1^Mt^*, a PCR-generated fragment (primers NJ66 and NJ67) containing *cco1^Mt^* (*Rv0654*; chromosome coordinates 750000 to 751505) was digested with AflII and SbfI, and then cloned into a pMLΔxylE backbone obtained by digestion of a derivative of pML1335-WCB2 [83] with AflII and SbfI. The construction placed *cco1^Mt^* under the control of the promoter *Pmyc1* [84]. To obtain pML-*fnr1*, a PCR-generated fragment (chromosome coordinates 4704636 to 4706005; primers NJ119 and NJ121) containing *fnr1* (*MKAN_RS20530*) and its promoter region was digested with AflII and MfeI, and then cloned into a pMLΔxylE backbone obtained by AflII-MfeI digestion of pML-*crtYd* with AflII and MfeI. The plasmid expressed *fnr1* from its native promoter. To construct pML-*desA3*, a PCR-generated fragment (chromosome coordinates 4705860 to 4707355; primers NJ122 and NJ120) containing *desA3* (*MKAN_RS20535*) and its promoter region was digested with AflII and MfeI, and then cloned into a pMLΔxylE backbone obtained by digestion of pML-*crtYd* with AflII and MfeI. The plasmid expressed *desA3* from its native promoter region. Additional information on the plasmids and primers used in this study are listed in Tables S2 and S3, respectively.

### 3.5. Pigmentation Phenotype Assessment

Exponentially growing cultures were normalized to an optical density (OD_600 nm_) of 0.7–0.8 by dilution with s7H9 and loaded in a 96-well plate (50 µL/well). The microcultures were then spotted onto duplicate s7H10 plates (15 cm diameter) using a 96-pin microplate replicator (Boekel Scientific, Feasterville, PA, United States). Each pair of inoculated plates was then incubated in the dark for 10–12 d. After the incubation, one of the plates in the pair was placed on a plexiglass sheet ~10 cm above a lightbox (cold white 40 W fluorescent light tubes) in a 37 °C incubator for ~3 h. After light exposure, the light-treated plate was returned to the dark incubator and incubated along with its untreated partner plate for ~2 additional days to allow for pigmentation development after the light treatment. After this incubation, the macrocolonies on the plates were imaged (10x magnification) using an Olympus SZX7 stereoscope and its associated CellSens Standard software (Olympus Life Science). In the case of *Ms* strains, cultures (OD_600_ of 0.5) were spotted (1 μL) onto s7H11 plates using a standard micropipette. The inoculated plates were incubated with or without light exposure as noted above for 3 d and the macrocolonies were imaged using the stereoscope. Where appropriate, the WT strain carried pML (empty vector, Hyg^R^ marker) or pCP0h (empty vector, Km^R^ and Hyg^R^ markers) so that the strain could be grown along with the Tn mutant strains in the same antibiotic-containing medium.

### 3.6. Preparation of RNA for RT-qPCR and RNA-Seq Analyses

Cultures grown in the dark to an OD_600 nm_ of 0.7 (15 mL, 250 mL flask) were incubated at 37 °C with manual agitation (swirling once every 5 min) 10 cm elevated above a lightbox (cold white 40 W fluorescent light tubes) for 20 min. Light-treated cultures and untreated control cultures (flasks wrapped in aluminum foil) were incubated side-by-side. After the incubation, the cultures were kept on ice for at least 15 min, cells were harvested by centrifugation, and cell pellets were frozen at −80 °C until needed, or immediately used for RNA isolation. Total RNA was isolated using the TRIzol Plus RNA Purification kit (Invitrogen) following the manufacturer’s instructions, except for the addition of a bead-beating step to improve cell lysis as previously described [38]. RNA was quantified using a NanoDrop 2000 Spectrophotometer (Thermo Fisher Scientific).

### 3.7. RT-qPCR Analysis

The experiments were performed using gene-specific primer pairs and the QuantiNova™ SYBR Green RT-PCR kit (Qiagen) following the manufacturer’s instructions. All primer pairs were optimized as reported [38]. One-step RT-qPCR reactions (10 μL, ~20 pg of RNA) were run in a Rotor-Gene Q real-time thermocycler (Qiagen) using 62 °C (10 s) for annealing/elongation temperature. Two-step RT-qPCR was used where strand-specific expression information was desired. In such cases, reaction mixtures were prepared only including the primer binding to the coding strand and the reverse transcription reaction was performed (10 min, 50 °C). Upon completion, the samples were chilled on ice, the second primer was added to them, and the samples were transferred to the thermocycler for the qPCR amplification (40 cycles; denaturation: 95 °C, 5 s; annealing/extension: 62 °C, 10 s). Data analysis and fold change determinations were performed using the 2^−ΔΔ*CT*^ method as reported and with normalization to the standard mycobacterial internal calibrator *sigA* (*MKAN_RS24220*) transcript [38]. When the expression level of a specific *Mk* gene was at or below the limit of detection, the detection limit value determined under our experimental conditions was used in place of the gene-specific expression for the fold change calculations. Statistical significance assessment was done using the unpaired, two-tailed *t*-test (Holm–Sidak method; alpha = 0.05) embedded in Prism v6.01 (GraphPad Software Inc., La Jolla, CA, United States).

### 3.8. RNA-Seq Analysis

Strand-specific library generation, sequencing on the Illumina NovaSeq platform, and data analysis for differentially expressed genes was carried out by Novogene (Sacramento, CA, USA) according to the company’s standard protocols (https://en.novogene.com/, accessed on 18 November 2022). Briefly, read mapping to the reference *Mk* genome was performed using Bowtie2 [86] and expression quantification using HTSeq [87]. For differential expression analysis, readcounts were normalized using the FPKM method (Fragments Per Kilobase of transcript sequence per Million base pairs sequenced), which takes into account the effects of both sequencing depth and gene length for counting of fragments [88]. Q-values were calculated after FPKM readcount normalization using a model-dependent *p*-value estimation and the DEGseq software [89]. Normalization was performed using the TMM normalization method. The Poisson distribution was used for the *p*-value estimation model and the BH FDR discovery method was used for FDR value estimation based on multiple hypothesis testing. For screening of differentially expressed genes, the cutoff values were set as |log2(FoldChange)| > 1 and q-value < 0.005. Volcano plots were created by Novogene and processed for publication using Adobe Illustrator (Adobe, San Jose, CA, United States). RNA-seq coverage plots were created from .bam files with Integrative Genomics Viewer (IGV, Version 2.8) [90] and processed for publication using Adobe Illustrator.

### 3.9. Routine Sequence Bioinformatics

DNA and protein sequence alignments were performed using BLAST or the DNASTAR Lasergene software package (DNASTAR Inc., Madison, WI, United States). Both DNASTAR Lasergene and Serial Cloner 2.6.1 (SerialBasics Software, (http://serialbasics.free.fr/Serial_Cloner.html, accessed on 18 November 2022) were used to assist DNA cloning, sequence analysis, and visualizations. Sequence alignment was done with the Clustal W algorithm embedded in the MegAlign application of the DNASTAR Lasergene software. Potential orthologues were identified using the BLAST-based Reciprocal Best Hits method [91]. Syntenic gene analysis was performed using SyntTax with standard parameters [92].

## 4. Conclusions

Our findings provide new insights into the genetic underpinnings of the biosynthesis and degradation of carotenes and the magnitude of the transcriptome remodeling in response to light in *Mk*. The identification of CrtR as a critical controller of the photochromogenic phenotype and CcoR as a predicted modulator of carotenoid pigment degradation opens new lines of research inquiry into the regulation of carotenoid metabolism in mycobacteria. Future studies aimed at investigating the nature of possible physiological small molecule ligands modulating the DNA binding activity of these regulators is warranted. Along this line, while light had no impact on the expression of genes involved in carotene degradation, the expression of the genes in the carotene biosynthetic pathway are postulated herein to be repressed by CrtR in the dark, but robustly induced by light exposure. It is tempting to speculate that, upon light exposure, photooxidation of some cellular metabolite(s) leads to generation of a CrtR ligand that acts as an inducer of carotenogenesis by inactivating the repressor function of the regulator. Of note, a recent study reported that production of the carotenoid decaprenoxanthin in *Corynebacterium glutamicum* is light-regulated and repressed by a CrtR homolog (CGTRNA_RS03160; 36% identity) [93]. Moreover, the in vitro DNA-binding activity of CGTRNA_RS03160 is perturbed by D-glyceraldehyde 3-phosphate and various isoprenoid pyrophosphates [94]. Although the molecular mechanism behind this in vitro observation is unknown and its physiological relevance unclear, the finding raises the possibility that the activity of *Mk* CrtR is modulated by a yet unidentified metabolite(s) linked to isoprenoid and/or carotenoid metabolism. Notably, the transcriptome changes in response to light in *Mk* include upregulation of genes involved in functions beyond carotenoid metabolism. It remains unknown whether CrtR plays a role in the regulation of these genes. The collection of *crtR* mutants generated herein will facilitate experiments to investigate these unknowns. Lastly, microbial carotenoids are known to play a wide range of roles, including in host-pathogen interaction and virulence [45,95,96,97]. The mutants generated in this work will also enable research to probe the relevance of carotenes/carotenogenesis in the virulence of *Mk* in infection models and the ex vivo fitness of the bacterium under different environmental stresses. Such endeavors might illuminate potential paths to new therapeutics.

## Figures and Tables

**Table 1:** *M. kansasii* genes differentially expressed in response to light exposure.

	Locus Tag ^1^ (Gene Name)	Predicted Gene Product Function ^2^	Dark Condition Readcount ^3^	Light Condition Readcount	Fold Change ^4^	Q-Value ^5^
CRT locus genes	*MKAN_RS10020* (*crtE*)	GGPP synthase	8	1278	160	1.5 × 10^−278^
	*MKAN_RS10025* (*crtI*)	Phytoene dehydrogenase	11	1771	161	0
	*MKAN_RS10030* (*crtB*)	Phytoene synthase	8	1124	141	7.9 × 10^−249^
	*MKAN_RS10035* (*crtYc*)	Lycopene cyclase	<1	29	221	3.6 × 10^−5^
	*MKAN_RS10040* (*crtYd*)	Lycopene cyclase	<1	88	334	1.5 × 10^−16^
	*MKAN_RS10045* (*mmpL1*)	MmpL family transporter	33	1339	41	0
	*MKAN_RS10050* (*crtR*)	MarR-type regulator	127	331	3	3.5 × 10^−18^
	*MKAN_RS10055* (*fni*)	Isopentenyl diphosphate isomerase	124	1007	8	3.4 × 10^−162^
Photolyase locus genes	*MKAN_RS22245* (*phrB*)	Deoxyribodipyrimidine photolyase	15	474	32	2.7 × 10^−106^
	*MKAN_RS22250* (*tspO*)	Tryptophan-rich sensory protein	9	258	29	5.1 × 10^−57^
	*MKAN_RS22255* (*mmpL2*)	MmpL family transporter	84	2277	27	0
Loci of unknown function	*MKAN_RS11600* (*mpk83*)	Fasciclin domain-containing protein	6	36	6	8.1 × 10^−4^
	*MKAN_RS11610* (*mpk70*)	Fasciclin domain-containing protein	21	64	3	1.0 × 10^−3^
	*MKAN_RS19770*	PE family protein	121	601	5	3.2 × 10^−71^

^1^ Locus tags and gene names for *MKAN_RS10025* (*crtI*) and *MKAN_RS10055* (*fni*) are as per NCBI genome annotation (NC_022663.1). Gene names other than *crtI* and *fni* were assigned herein as per sequence homology and/or orthology analysis. ^2^ Functions listed are as per NCBI genome annotation pipeline. ^3^ Readcounts and Q-values were determined as described in Materials and Methods. Readcounts are rounded to the nearest whole number. In the case of *MKAN_RS10035* (*crtYc*) and *MKAN_RS10040* (*crtYd*), the calculated readcounts for the dark condition were below 1. ^4^ Fold changes are rounded to the nearest whole number and calculated based on the rounded readcounts, except in the case of *MKAN_RS10035* (*crtYc*) and *MKAN_RS10040* (*crtYd*), where the decimal (not rounded) number was used. ^5^ Values below 2.2251 × 10^−308^ are shown as 0.

## Data Availability

Not applicable.

## Supplementary Materials

The following supporting information can be downloaded at: https://www.mdpi.com/article/10.3390/pathogens12010086/s1, Figure S1: Modeled probability (*p*) of no transposon insertion in an *M. kansasii* gene with *n* number of TA sites in our library of ~150,000 transposon mutants; Figure S2: RNA-seq analysis of selected *M. kansasii* genes in the wild-type strain with or without light treatment; Figure S3: Pairwise amino acid sequence identity matrix of all fifteen MmpL protein paralogues annotated for *M. kansasii*; Figure S4: *M. kansasii* develops red speckles when grown with continuous light exposure; Figure S5: Overlaps of CRT locus genes; Figure S6: Volcano plot of differentially expressed genes after light exposure; Figure S7: Southern blot analysis of *M. kansasii* mutants; Table S1: Transposon insertion sites in *M. kansasii* isolates; Table S2: Mycobacterial gene expression plasmids used in this study; Table S3: Primers used in the study; Supplementary HAGRID method for genomic DNA isolation.
