# Supplementary material for: Genetic Underpinnings of Carotenogenesis and Light-Induced Transcriptome Remodeling in the Opportunistic Pathogen Mycobacterium kansasii
Source: Pathogens. 2023 Jan 5;12(1):86. doi: 10.3390/pathogens12010086 (PMC9861118; doi:10.3390/pathogens12010086)
Supplement: Supplementary file 1 [file pathogens-12-00086-s001.zip › Supplementary Figure Captions_Janisch et al.pdf]

Janisch et al. Supplementary Figure Captions

Figure S1. Modeled probability (*p*) of no transposon insertion in an *M. kansasii* gene with *n* number of TA sites in our library of ~150,000 transposon mutants. Assuming that the probability of a transposon insertion is uniform across all TA sites in the genome and independent across mutants, the chance of no transposon insertion in a given gene can be modeled with the exponential function $p = e^{-n*x/g}$, where *n* = the number of TA sites in the gene, *x* = number of transposon mutants in the library, and *g* = the number of TA sites in the genome of *M. kansasii*. The equation specific for our *M. kansasii* library is shown.

Figure S2. RNA-seq analysis of selected *M. kansasii* (*Mk*) genes in the wild-type (WT) strain with or without light treatment. 'Light' and 'Dark' designations denote cultures with and without 20-min light treatment, respectively. Gene names and locus tags are assigned as noted in the manuscript proper. Genome coordinates of the chromosomal segments displayed are indicated. (**a**) CRT locus genes. The transcription start site at the 5' end of the locus (TSS1) is indicated in the top panel. The bottom panel shows the zoomed-in TSS1 region. (**b**) *crtR* and *fni* region. The transcription start sites upstream of *crtR* (TSS2 and TSS3) are indicated in the top panel. Note that the readcount scale for 'Light' and 'Dark' samples are set to different values to allow for a meaningful visualization. The middle and bottom panels show the zoomed-in TSS2 and TSS3 regions, respectively. Both genes show significant transcription in the dark and are further induced by light. (**c**) *MKAN_RS31895*. (**d**) $cco1^{Mk}$ (top panel) and $ccoR^{Mk}$ (bottom panel). (**e**) FD locus genes. (**f**) Photolyase locus genes. (**g**) *mpk83-dipZ-mpk70* locus. (**h**) *MKAN_RS19770*. (**i**) *MKAN_RS02680* and *MKAN_RS17995*.

Figure S3. Pairwise amino acid sequence identity matrix of all fifteen MmpL protein paralogues annotated for *M. kansasii*. The closest paralogues of the MmpL1 protein encoded in the CRT locus (MKAN_RS10045, yellow box) are MKAN_RS07110, MKAN_RS21020, MKAN_RS21440 (red print). Amino acid identity percentages of the best three paralogue pairs (58-62% identity) are highlighted (red boxes). Sequence alignment was done with the Clustal W algorithm embedded in the MegAlign application of the DNASTAR Lasergene software.

Figure S4. *M. kansasii* develops red speckles when grown with continuous light exposure. Macrocolonies of the wild-type strain were grown in the dark for 18 days ('Dark' column), in the dark for 15 days, exposed to light for 3 hours, and incubated in the dark for an additional 3 days

(‘Light^{3h}’ column), or with continuous light exposure for 18 days (‘Light^{c}’ column). Top row scale bar = 2 mm. Bottom row scale bar = 0.2 mm. Results shown are representative of three independent experiments. Macrocolonies were imaged with an Olympus SZX7 stereo microscope (Olympus Life Science, Waltham, MA, United States). The magnification factor is indicated on the left.

Figure S5. Overlaps of CRT locus genes. Image adapted from the *M. kansasii* genome page (NCBI reference sequence: NC_022663.1).

Figure S6. Volcano plot of differentially expressed genes after light exposure. Fourteen genes were found to be upregulated (red dots) in response to light exposure. The rest of the genes (blue dots) did not show differential expression. The locus tags of the upregulated genes are indicated. CRT locus, yellow boxes; *mmpL2*-*tspO*-*phrB* locus, green boxes; *mpk70*-*mpk83* locus, orange boxes; PE protein family gene, cyan box. Cultures of *M. kansasii* wild-type grown in the dark were split into two. One of the cultures was then subjected to a 20-min light treatment, while the other was kept shielded from light. Genes with fold changes greater than two (log2>1) and a q-value below 0.005 ($-\log_{10}$(qval)>2.3) were considered differentially expressed.

Figure S7. Southern blot analysis of *M. kansasii* mutants. The assemblage of southern blot images shown includes results for representative mutants selected for extended characterization. The analysis verified the presence of single transposon insertions in the mutants. Lane wt, wild-type genomic control digest. Blot images grouped are from the same blot where additional samples were cut out of the image for clarity. Lane M, molecular weight marker (Roche DNA Molecular Weight Marker VII, DIG-labeled). bp, base pairs. Arrows mark positions of observed hybridization bands. Observed hybridizing fragment sizes are in agreement with expected fragments resulting from AatII digestion (*i.e.*, genomic fragment size + Tn fragment size).
