# Supplementary material for: Genetic Underpinnings of Carotenogenesis and Light-Induced Transcriptome Remodeling in the Opportunistic Pathogen Mycobacterium kansasii
Source: Pathogens. 2023 Jan 5;12(1):86. doi: 10.3390/pathogens12010086 (PMC9861118; doi:10.3390/pathogens12010086)
Supplement: Supplementary file 1 [file pathogens-12-00086-s001.zip › Supplementary HAGRID Method_Janisch et al.pdf]

HAGRID Protocol For Genomic DNA Isolation

The HAGRID (Hot Phenol And Glass bead protocol for Rapid Isolation of mycobacterial DNA) protocol is derived from the methods reported by Belisle et al. 1998 and Billi et al. 1998 [79,80]. Changes include the reduction of initial wash steps of the cell pellet, addition of a heat inactivation step, bead beating in phenol:chloroform:isoamyl alcohol (P:C:I), rather than in buffer as in Belisle et al. 1998 or phenol as in Billi et al. 1998, complete elimination of separate steps of enzymatic digest (proteinase K, lysozyme), P:C:I and chloroform extraction, and the addition of steps for better resuspension of hard-to-dissolve genomic DNA pellets.

A) REAGENTS AND MATERIALS TO BE PREPARED BEFOREHAND

1) TET (Tris-EDTA-Triton) Extraction Buffer (200 mL)

Component	Stock Concentration	Volume Added	Final Concentration
Tris-HCl, pH 8	1 M	2 mL	10 mM
EDTA, pH 8	0.05 M	4 mL	1 mM
Triton X-100	-	20 µL	0.01 %
Distilled water (dH_2O)	-	Up to final volume of 200 mL	-

2) 3 M Sodium Acetate (10 mL, pH 5.2)

For 10 mL: dissolve 2.46 g sodium acetate (MW = 82.03 g/mol) in 8 mL dH_2O. Adjust the pH of the solution to 5.2 with glacial acetic acid (10 M). Bring up to a total volume of 10 mL with dH_2O.

3) Phenol:chloroform:isoamyl alcohol (P:C:I) (25:24:1, v/v/v) (from VWR International, LLC, Radnor, PA, United States or equivalent supplier).

Note: make sure to add the pH-increasing buffer (provided with the P:C:I product) to P:C:I as per product instructions to bring the pH of the mixture to 8 before first use.

4) Microcentrifuge Tubes

Note: initial steps involving live bacteria and P:C:I need to be performed using phenol/chloroform-resistant (polypropylene) 1.5 mL microcentrifuge tubes (preferably conical bottom) with a screw-

cap and a rubber o-ring for tight sealing. Subsequent steps (isopropanol precipitation and ethanol wash) are performed in nuclease-free, 1.5 mL microcentrifuge tubes (preferably conical bottom).

B) STEPWISE PROTOCOL

Note: if working with BSL-2 organisms (e.g., *M. kansasii, M. abscessus*), perform steps 1 – 8 in a biosafety cabinet and prevent aerosolization of the pathogen.

1. Prepare bead-beating tubes by adding ~500 µL 0.1-0.5 mm glass or zirconia beads and 500 µL P:C:I to a 1.5 mL microcentrifuge tube with a screw cap and rubber o-ring. Note: do not use regular snap-cap microcentrifuge tubes.

2. Centrifuge 1.5-5 mL of culture to obtain cell pellets in 1.5 mL or 15 mL tubes (not in the tubes containing beads and P:C:I). Note: cell pellets can be processed immediately or frozen at -20°C for later processing.

3. Re-suspend each pellet in 500 µL of TET extraction buffer by pipetting.

4. Transfer the cell suspension to the microcentrifuge tubes containing beads and 500 µL P:C:I (see step 1). Make sure the tubes are tightly closed.

5. Place the microcentrifuge tubes in a cell disruptor (Disruptor Genie, VWR) and disrupt for 1 min.

   The following steps a.-c. are optional, but may aid killing of bacteria and/or increase DNA yields by improving bacterial lysis.

   a. Place the microcentrifuge tubes in a heat block at 65°C for 5 min.
   b. Transfer the microcentrifuge tubes to the cell disruptor and disrupt for 1 min.
   c. Repeat steps a. and b. once more.

6. Centrifuge the microcentrifuge tubes (13,000-15,000 rpm or 15,000-21,000 x g for 2 min at room temperature in a benchtop centrifuge). Note: do not discard the supernatant.

7. Meanwhile, label 1.5 mL microcentrifuge tubes (regular tubes) and add 50 µL of 3 M sodium acetate (NaOAc, pH 5.2) and 500 µL of 100% isopropanol to each of them.

8. Carefully recover only the aqueous layer (top layer) of the P:C:I extraction and add it to the microcentrifuge tubes containing NaOAc and isopropanol (see step 7). Avoid taking the cloudy interphase and the lower phase. (You may need to leave some of the upper phase behind, that is fine.) Vortex the tubes thoroughly.

9. Centrifuge the microcentrifuge tubes at 13,000-15,000 rpm (or max speed of a tabletop centrifuge) and 4°C for 15 min.

10. Carefully remove the supernatant using a clean pipette tip connected to a vacuum line with a solvent trap. Alternatively, use a pipette to remove the supernatant. When removing the supernatant, be careful not to disturb the pellet.

11. To remove residual organic solvents, add 750 µL 70% EtOH, vortex, centrifuge at 13,000-15,000 rpm (or max speed of a tabletop centrifuge) and 4°C for 10 min, and suction off the supernatant thoroughly. Be careful not to disturb the pellet.

12. Place tubes with lids open at 50°C for ~10 min in a chemical fume hood to let residual EtOH/organic solvents evaporate. Do not let the tubes sit for much longer as the genomic DNA pellet will dry onto the plastic and become hard to dissolve.

13. Meanwhile, pre-warm nuclease-free water to 40-50°C on a heat block.

14. Resuspend the DNA pellet by adding 50 µL of warm nuclease-free water, vortex/disrupt for 1-2 min, and centrifuge at 13,000-15,000 rpm (or maximum speed of a tabletop centrifuge) for 1 min. 30 sec in a water-bath sonicator at room-temperature can help further resuspend pellets.

15. Store the isolated genomic DNA at 4°C for short-term storage or at -20°C for long-term storage.
