# Supplementary material for: Genetic Underpinnings of Carotenogenesis and Light-Induced Transcriptome Remodeling in the Opportunistic Pathogen Mycobacterium kansasii
Source: Pathogens. 2023 Jan 5;12(1):86. doi: 10.3390/pathogens12010086 (PMC9861118; doi:10.3390/pathogens12010086)
Supplement: Supplementary file 1 [file pathogens-12-00086-s001.zip › Table S2_Janisch et al.pdf]

Table S2. Mycobacterial gene expression plasmids used in this study

Plasmid name	Characteristics	Source
pML	pML1335 derivative lacking the *xylE* marker and used as empty vector control	[85]
pML-*crt*	pML derivative carrying *MKAN_RS10020* to *MKAN_RS10050*, including the upstream intergenic region containing P1 promoter region	This study
pML-*crtE*	pML derivative carrying P1 promoter region-*MKAN_RS10020* fragment	This study
pML-*crtI*	pML derivative carrying P1 promoter region + *MKAN_RS10025* coding region fragment	This study
pML-*crtB*	pML derivative carrying P1 promoter region + *MKAN_RS10030* coding region fragment	This study
pML-*crtYc*	pML derivative carrying P1 promoter region + *MKAN_RS10035* coding region fragment	This study
pML-*crtYd*	pML derivative carrying P1 promoter region + *MKAN_RS10040* coding region fragment	This study
pML^{myc}-*crtYd*	pML derivative carrying Pmyc1 promoter + *MKAN_RS10040* fragment	This study
pML-*crtR*	pML derivative carrying P3 promoter region-*MKAN_RS10050* fragment	This study
pML-$cco1^{Mk\text{-}part}$-$ccoR^{Mk}$	pML derivative carrying *MKAN_RS18575-MKAN_RS18580* (partial)	This study
pML^{myc}-$cco1^{Mk}$	pML derivative carrying Pmyc1 promoter-*MKAN_RS18580* fragment	This study
pML^{myc}-$cco1^{Mt}$	pML derivative carrying Pmyc1 promoter-*Rv0654* fragment	This study
pML-$cco1^{Mk}$	pML derivative carrying native promoter region-*MKAN_RS18580* fragment	This study
pML-$cco1^{Mt}$	pML derivative carrying native promoter region-*Rv0654* fragment	This study
pML-$ccoR^{Mk}$	pML derivative carrying native promoter region-*MKAN_RS18575* fragment	This study
pML-$ccoR^{Mt}$	pML derivative carrying native promoter region-*Rv0653c* fragment	This study
pML-*fnr1*	pML derivative carrying native promoter region-*MKAN_RS20530* fragment	This study
pML-*desA3*	pML derivative carrying native promoter region-*MKAN_RS20535* fragment	This study
pCP0h	pCP0 derivative carrying its native kanamycin resistance cassette and the hygromycin resistance cassette (HpaI-PmeI fragment) from pML1335 inserted into the DraI restriction site of the vector	This study
