# Supplementary material for: Genetic Underpinnings of Carotenogenesis and Light-Induced Transcriptome Remodeling in the Opportunistic Pathogen Mycobacterium kansasii
Source: Pathogens. 2023 Jan 5;12(1):86. doi: 10.3390/pathogens12010086 (PMC9861118; doi:10.3390/pathogens12010086)
Supplement: Supplementary file 1 [file pathogens-12-00086-s001.zip › Table S3_Janisch et al.pdf]

Table S3. Primers used in the study

Name	Sequence (5' → 3')	Features
**Southern blot primers**		
GG1<br>GG2	GACTGGCTGCTATTGGGCGAAG<br>AGAAGGCGATAGAAGGCGATG	Primers for PCR-based generation of Tn-specific probe, ϕMycoMarT7 (ϕMMT7) transposon complementary sequence (NCBI reference sequence AF411123.1) location 1467-1978
**Complementation construct primers**		
NJ1	AAATTATAAGATAACCAGTTCCTGGTACC TCACCA	Forward primer for the generation of pML-*crt*, containing: PsiI site (underlined) and *Mycobacterium kansasii* (*Mk*) genomic complementary sequence starting at NC_022663.1 (NCBI reference sequence) genomic location 2342585
NJ2	AAACAATTGGTTCATCGAGGAGCTTCGC ATCT	Reverse primer for the generation of pML-*crt* and pML-*crtR,* containing: MfeI site (underlined) and *Mk* genomic complementary sequence starting at NC_022663.1 genomic location 2350376
NJ3	AAATTATAAGAGACCGGTGAAGCCGGTG AGG	Forward primer for the generation of pML-*crtR,* containing: PsiI site (underlined) and *Mk* genomic complementary sequence starting at NC_022663.1 genomic location 2349511
NJ46	AAATCATGA**GCCCGCCTAATGGCGGGC TTTTTTTTGAATTC**TCAACCGAAAGCGGA GCGCTGCGACTT	Forward primer for the generation of pML-*cco1*$^{Mk\text{-}part}$-*ccoR*^{Mk} containing: BspHI site (underlined), $tt_{trp}A$+ transcriptional terminator from pML1335 (bold) [81], EcoRI site (bold and underlined) and *Mk* genomic complementary sequence starting at NC_022663.1 genomic location 4262321
NJ47	AAACAATTG**GGCCGGCCC**TTGGGGTTG CGGTTCATC	Reverse primer for the generation of pML-*cco1*$^{Mk\text{-}part}$-*ccoR*^{Mk} containing: MfeI site (underlined), FseI site (bold and underlined) and *Mk* genomic complementary sequence starting at NC_022663.1 genomic location 4263914
NJ112	TGGCGGGCTTTTTTTTCTTAAGGATAACC AGTTCCTGGTACCTCACCA	Forward primer for the generation of pML-*crtE*, and for the generation of the carotenoid biosynthesis (CRT) locus promoter P1 fragment of pML-*crtI*, pML-*crtB,* pML-*crtYc* and pML-*crtYd*, containing: AflII site (underlined) and *Mk* genomic complementary sequence starting at NC_022663.1 genomic location 2342585
NJ102	GGCTCGCCAAGAAGCGGAATCGACGGT GGGT	Reverse primer for the generation of the CRT locus promoter P1 fragment of pML-*crtI*, pML-*crtB,* pML-*crtYc* and pML-*crtYd* containing: *Mk* genomic complementary sequence starting at NC_022663.1 genomic location 2343093
NJ101	AAACAATTGTCATGCGGTGCGCTCCACG	Reverse primer for the generation of pML-*crtE* containing: MfeI site (underlined), *Mk* genomic complementary sequence starting at NC_022663.1 genomic location 2344128
NJ103	**GCTACCGATCACCCACCGTCGATTCCG CTTCTTGGCGAGCCATG**CGGACCATCC AGGGAC	Forward primer for the generation of pML-*crtI* containing: *Mk* genomic complementary sequence from NC_022663.1 genomic location 2343053 to 2343096 (3'

		of P1 promoter, bold) fused to sequence starting at NC_022663.1 genomic location 2344145 (5' of *crtI)*
NJ104	**CATTAGGCGGGCT**CAATTGTCATGACATCTGCGCTTTCACGTCGA	Reverse primer for the generation of pML-*crtI* containing: pML1335Δ*xylE* sequence (bold), MfeI site (underlined), *Mk* genomic complementary sequence starting at NC_022663.1 genomic location 2345683
NJ105	**ATCACCCACCGTCGATTCCGCTTCTTGGCGAGCCATG**ATTCACAACGAGTTAGACGCCGC	Forward primer for the generation of pML-*crtB* containing: *Mk* genomic complementary sequence from NC_022663.1 genomic location 2343060 to 2343096 (3' of P1 promoter, bold) fused to sequence starting at NC_022663.1 genomic location 2345683 (5' of *crtB)*
NJ106	**CATTAGGCGGGCT**CAATTGTCATGCGGCACCCGGCTC	Reverse primer for the generation of pML-*crtB* containing: pML1335Δ*xylE* sequence (bold), MfeI site (underlined), *Mk* genomic complementary sequence starting at NC_022663.1 genomic location 2346642
NJ107	**GATCACCCACCGTCGATTCCGCTTCTTGGCGAGCCATG**AACGATCACTGGCAATACCTGC	Forward primer for the generation of pML-*crtYc* containing: *Mk* genomic complementary sequence from NC_022663.1 genomic location 2343059 to 2343096 (3' of P1 promoter, bold) fused to sequence starting at 2346642 (5' of *crtYc)*
NJ108	**CATTAGGCGGGCT**CAATTGTCATCGGCGGGTGAGCCG	Reverse primer for the generation of pML-*crtYc* containing: pML1335Δ*xylE* sequence (bold), MfeI site (underlined), *Mk* genomic complementary sequence starting at NC_022663.1 genomic location 2346963
NJ109	**ACCGATCACCCACCGTCGATTCCGCTTCTTGGCGAGCCATG**ACCGGGCTGGGATATACGC	Forward primer for the generation of pML-*crtYd* containing: *Mk* genomic complementary sequence from NC_022663.1 genomic location 2343056 to 2343096 (3' of P1 promoter, bold) fused to sequence starting at 2346962 (5' of *crtYd)*
NJ110	**CATTAGGCGGGCT**CAATTGTCATCGACGCGCTCGCGG	Reverse primer for the generation of pML-*crtYd* containing: pML1335Δ*xylE* sequence (bold), MfeI site (underlined), *Mk* genomic complementary sequence starting at NC_022663.1 genomic location 2347270
NJ147	AAACTTAAG**GAAGGAGATATACAT**ATGACCGGGCTGGGATATACGC	Forward primer for the generation of pML^{myc}-*crtYd*, containing: AflII site (underlined), ribosome binding site (bold) and spacer (bold, underlined) from pSE100 and *Mk* genomic complementary sequence starting at NC_022663.1 genomic location 2346959
NJ145	AAACCTGCAGGTCATCGACGCGCTCGCGG	Reverse primer for the generation of pML^{myc}-*crtYd,* containing: SbfI site (underlined) and *Mk* genomic complementary sequence starting at NC_022663.1 genomic location 2347270
NJ64	AAACTTAAG**GAAGGAGATATACAT**GTGACGACAATGCGAACTGCC	Forward primer for the generation of pML^{myc}-*cco1*^{Mk}, containing: AflII site (underlined), ribosome binding site (bold) and spacer (bold, underlined) from pSE100 and *Mk* genomic complementary sequence starting at NC_022663.1 (NCBI reference sequence) genomic location 4263096
NJ65	AAACCTGCAGGTCAAGGAGTCGCCGGTGC	Reverse primer for the generation of pML^{myc}-*cco1*^{Mk}*,* containing: SbfI site (underlined) and *Mk* genomic complementary sequence starting at NC_022663.1 genomic location 4264616
NJ66	AAACTTAAG**GAAGGAGATATACAT**ATGACCACCGCACAAGCC	Forward primer for the generation of pML^{myc}-*cco1*^{Mt}, containing: AflII site (underlined), ribosome binding site (bold) and spacer (bold, underlined) from pSE100 and

		Mycobacterium tuberculosis (*Mt*) genomic complementary sequence starting at NC_000962.3 (NCBI reference sequence) genomic location 750000
NJ67	AAACCTGCAGGTCAGGTGGTCGGCGCCCA	Reverse primer for the generation of pML^{myc}-*cco1*^{Mt}, containing: SbfI site (underlined) and *Mt* genomic complementary sequence starting at NC_000962.3 genomic location 751505
NJ111	AAACTTAAG**TCA**GCGGGTTTGCAGGGCATCGGGT	Forward primer for the generation of pML-*cco1*^{Mk}, containing: AflII site (underlined), stop codon (bold) and *Mk* genomic complementary sequence starting at NC_022663.1 genomic location 4262909
NJ78	AAACAATTGTCAAGGAGTCGCCGGTGCCC	Reverse primer for the generation of pML-*cco1*^{Mk} containing: MfeI site (underlined), and *Mk* genomic complementary sequence starting at NC_022663.1 genomic location 4264616
NJ79	AAACTTAAGCCTTGCCATGTTCTCACCAAGGGCG	Forward primer for the generation of pML-*cco1*^{Mt}, containing: AflII site (underlined) and *Mt* genomic complementary sequence starting at NC_000962.3 genomic location 749930
NJ80	AAACAATTGTCAGGTGGTCGGCGCCCAG	Reverse primer for the generation of pML-*cco1*^{Mt} containing: MfeI site (underlined), and *Mt* genomic complementary sequence starting at NC_000962.3 genomic location 751505
NJ56	AAATCATGA**GCCCGCCTAATGGCGGGCTTTTTTTTCTTAAGT**CAACCGAAAGCGGAGCGC	Forward primer for the generation of pML-*ccoR*^{Mk} containing: BspHI site (underlined), *tt*$_{trp}$*A+* transcriptional terminator from pML1335 (bold), AflII site (bold, underlined) and *Mk* genomic complementary sequence starting at NC_022663.1 genomic location 4262322
NJ57	AAACAATTG**GGCCGGCC**TTCGCATTGTCGTCACGGCCGA	Reverse primer for the generation of pML-*ccoR*^{Mk} containing: MfeI site (underlined), FseI site (bold) and *Mk* genomic complementary sequence starting at NC_022663.1 genomic location 4263111
NJ58	AAA**TCATGA**GCCCGCCTAATGGCGGGCTTTTTTTT**CTTAAG**TCAAGTAGCTCTGCCCAGC	Forward primer for the generation of pML-*ccoR*^{Mt} containing: BspHI site (bold), *tt*$_{trp}$*A+* transcriptional terminator from pML1335 (underlined), AflII site (bold and underlined) and *Mt* genomic complementary sequence starting at NC_000962.3 genomic location 749235
NJ59	AAACAATTG**GGCCGGCC**GGCGGCTTGTGCGGTGGTCAT	Reverse primer for the generation of pML-*ccoR*^{Mt} containing: MfeI site (underlined), FseI site (bold) and *Mt* genomic complementary sequence starting at NC_000962.3 genomic location 750020
NJ119	AAACTTAAGCCGCAGACGCCTCGAAGCCT	Forward primer for the generation of pML-*fnr1*, containing: AflII site (underlined), and *Mt* genomic complementary sequence starting at NC_000962.3 genomic location 4704636
NJ121	AAACAATTGTTAGATGTCGACCACACAATCGCCCGAGGCA	Reverse primer for the generation of pML-*fnr1* containing: MfeI site (underlined), and *Mt* genomic complementary sequence starting at NC_000962.3 genomic location 4706005
NJ122	AAACTTAAGGCCGGATGGGGATCTGTCAATCGT	Forward primer for the generation of pML-*desA3*, containing: AflII site (underlined), and *Mt* genomic complementary sequence starting at NC_000962.3 genomic location 4705860

NJ120	AAACAATTGTCAGGCCGCCAGGTCCGCT	Reverse primer for the generation of pML-*desA3* containing: MfeI site (underlined), and *Mt* genomic complementary sequence starting at NC_000962.3 genomic location 4707355
**Plasmid rescue method primers**		
CB2	GAGATAGGGTTGAGTGTTGTTCC	Reverse (relative to the kanamycin-resistance cassette (Km^R)), outward-facing sequencing primer starting at ϕMMT7 transposon complementary sequence (NCBI reference sequence AF411123.1) location 84; see Budell et al. 2020 [38]
SR2b	CGCTTCCTCGTGCTTTACGGTATCGC	Forward (relative to Km^R), outward-facing sequencing primer starting at ϕMMT7 transposon complementary sequence (AF411123.1) location 1914; see Budell et al. 2020 [38]
**AP-PCR method primers**		
TR-1	GGATCTGGTTCCGCGCACATT	Reverse, transposon primer for the first round of arbitrarily primed PCR (AP-PCR), starting at ϕMMT7 transposon complementary sequence (AF411123.1) location 240
ARB-1b	GCGCTCACAGCAGTCGAGTCGAGANNN NNNNNNN**CGGCG**	Forward, arbitrary primer for the first round of AP-PCR, containing an adapter sequence, a stretch of random nucleotides (N) and a pentameric sequence common in the *Mk* genome (bold)
TR-2	CCAATAGGCCGAAATCGGCAAAATCCCT	Reverse, transposon primer for the second round of AP-PCR, starting at ϕMMT7 transposon complementary sequence (AF411123.1) location 133; This primer is also used for sequencing of PCR products.
ARB-2	GCGCTCACAGCAGTCGAGTCGAGA	Forward primer for the second round of AP-PCR, binding to the adapter sequence of the PCR fragment created with primer ARB-1b in the first round of AP-PCR
**Locus-targeted PCR method primers**		
NJ1	AAATTATAAGATAACCAGTTCCTGGTACC TCACCA	Forward (NJ1) and reverse (NJ37) primers for fragment 1 of the *Mk* carotenoid biosynthesis locus (CRT locus), NC_022663.1 genomic complementary sequence location 2342585 – 2344674, yielding a 2099 bp fragment
NJ37	ATGGCGTCCCAGTTGCGGAAT	
NJ8	GGCTGTACCGCACCGAAAT	Forward (NJ8) and reverse (NJ38) primers for fragment number 2 of the *Mk* CRT locus, NC_022663.1 genomic complementary sequence location 2344554 – 2346653, yielding a 2100 bp fragment
NJ38	CCAGTGATCGTTCATGCGGCA	
NJ13	GACGATTTCGAGGTGTTCAGTCA	Forward (NJ13) and reverse (NJ39) primers for fragment number 3 of the *Mk* CRT, NC_022663.1 genomic complementary sequence location 2346499 – 2348334, yielding a 1836 bp fragment
NJ39	CCGTACGTTGAGCGCATCGA	
NJ17	TCGCGGTCGTGGTCTTTT	Forward (NJ17) and reverse (NJ2) primers for fragment number 3 of the *Mk* CRT, NC_022663.1 genomic complementary sequence location 2348240 – 2350376, yielding a 2146 bp fragment
NJ2	AAACAATTGGTTCATCGAGGAGCTTCGC ATCT	

NJ156	GGCCCAGCACGCAATCACCAGGA	Forward (NJ156) and reverse (NJ157) primers for a fragment of the *Mk* CRT locus containing *MKAN_RS10035* and *MKAN_RS10040* (*crtYc and crtYd*), NC_022663.1 genomic complementary sequence location 2346366 – 2347539, yielding a 1174 bp fragment
NJ157	GCCACTGTCCGAGTTCACCAGCAGCA	
**RT-qPCR primers**		
NJ81	CCAGCCCCGTACTCTCGACGA	Forward (NJ81) and reverse (CB11, see Budell et al. 2020 [38]) RT-qPCR primers for *MKAN_RS24220* (*sigA*), amplifying *Mk* NC_022663.1 genomic complementary sequence location 5589256 – 5589392, yielding a 133-bp amplicon
CB11	ATCCAGGTAGTCGCGCAGGACT	
NJ82	CTGCGGACCGTCCTCGACAT	Forward (NJ82) and reverse (NJ83) RT-qPCR primers for *MKAN_RS10020* (*crtE*), amplifying *Mk* NC_022663.1 genomic complementary sequence location 2343625 – 2343758, yielding a 133-bp amplicon
NJ83	ATCGGCACCGTAGAGCCCGA	
NJ148	CTCGACGTTGTCACCCCGCT	Forward (NJ148) and reverse (NJ149) RT-qPCR primers for *MKAN_RS10025* (*crtI*), amplifying *Mk* NC_022663.1 genomic complementary sequence location 2345402 – 2345530, yielding a 129-bp amplicon
NJ149	ATTGTCGACGCCGCGAACCA	
NJ150	GGCAACCGCTTTGACGCTGTACT	Forward (NJ150) and reverse (NJ151) RT-qPCR primers for *MKAN_RS10030* (*crtB*), amplifying *Mk* NC_022663.1 genomic complementary sequence location 2346450 - 2346584, yielding a 135-bp amplicon
NJ151	CAGGCTTGGATCAGTCCGGCT	
NJ152	GTGGCCGTTCCGTTTCGGGT	Forward (NJ152) and reverse (NJ153) RT-qPCR primers for *MKAN_RS10035* (*crtYc*), amplifying *Mk* NC_022663.1 genomic complementary sequence location 2346843 - 2346954, yielding a 112-bp amplicon
NJ153	GGGTGAGCCGATCGAGAATCGT	
NJ154	AAACGACGGGCCTGCGGTT	Forward (NJ154) and reverse (NJ155) RT-qPCR primers for *MKAN_RS10040* (*crtYd*), amplifying *Mk* NC_022663.1 genomic complementary sequence location 2347158 - 2347277, yielding a 120-bp amplicon
NJ155	GGCGACTTCATCGACGCGCT	
NJ84	ACCTCACGTTTGCCGGTGGT	Forward (NJ84) and reverse (NJ85) RT-qPCR primers for *MKAN_RS10045* (*mmpL1*), amplifying *Mk* NC_022663.1 genomic complementary sequence location 2348923 - 2349052, yielding a 130-bp amplicon
NJ85	AAGCCGCTGTGAGGGAAAGCA	

NJ86	GCAGCATGGAGTTGGCTCACGA	Forward (NJ86) and reverse (NJ87) RT-qPCR primers for *MKAN_RS10050* (*crtR*), amplifying *Mk* NC_022663.1 genomic complementary sequence location 2350031 - 2350168, yielding a 138-bp amplicon
NJ87	CAAATGCAGACATCCCCGCGATCA	
NJ89	TCGCTACGGTGAACTGCGCT	Forward (NJ89) and reverse (NJ88) RT-qPCR primers for *MKAN_RS10055* (*fni*), amplifying *Mk* NC_022663.1 genomic complementary sequence location 2350482 - 2350593, yielding a 134-bp amplicon
NJ88	CGTCCATCCCGGTGCGGATA	
NJ91	CCATCGAGCGGCCGCATATGT	Forward (NJ91) and reverse (NJ90) RT-qPCR primers for *MKAN_RS18575* (*ccoR^Mk^*), amplifying *Mk* NC_022663.1 genomic complementary sequence location 4262594 - 4262724, yielding a 131-bp amplicon
NJ90	ATGCGCGAAGCTGGGATGGT	
NJ92	CCGCTCAACGCCTACACCGA	Forward (NJ92) and reverse (NJ93) RT-qPCR primers for *MKAN_RS18580* (*cco1^Mk^*), amplifying *Mk* NC_022663.1 genomic complementary sequence location 4264038 - 4264165, yielding a 128-bp amplicon
NJ93	CGATCCAGTGACGGACGGGTGT	
