# Supplementary figures and images for: Genetic Underpinnings of Carotenogenesis and Light-Induced Transcriptome Remodeling in the Opportunistic Pathogen Mycobacterium kansasii
Source: Pathogens. 2023 Jan 5;12(1):86. doi: 10.3390/pathogens12010086 (PMC9861118; doi:10.3390/pathogens12010086)

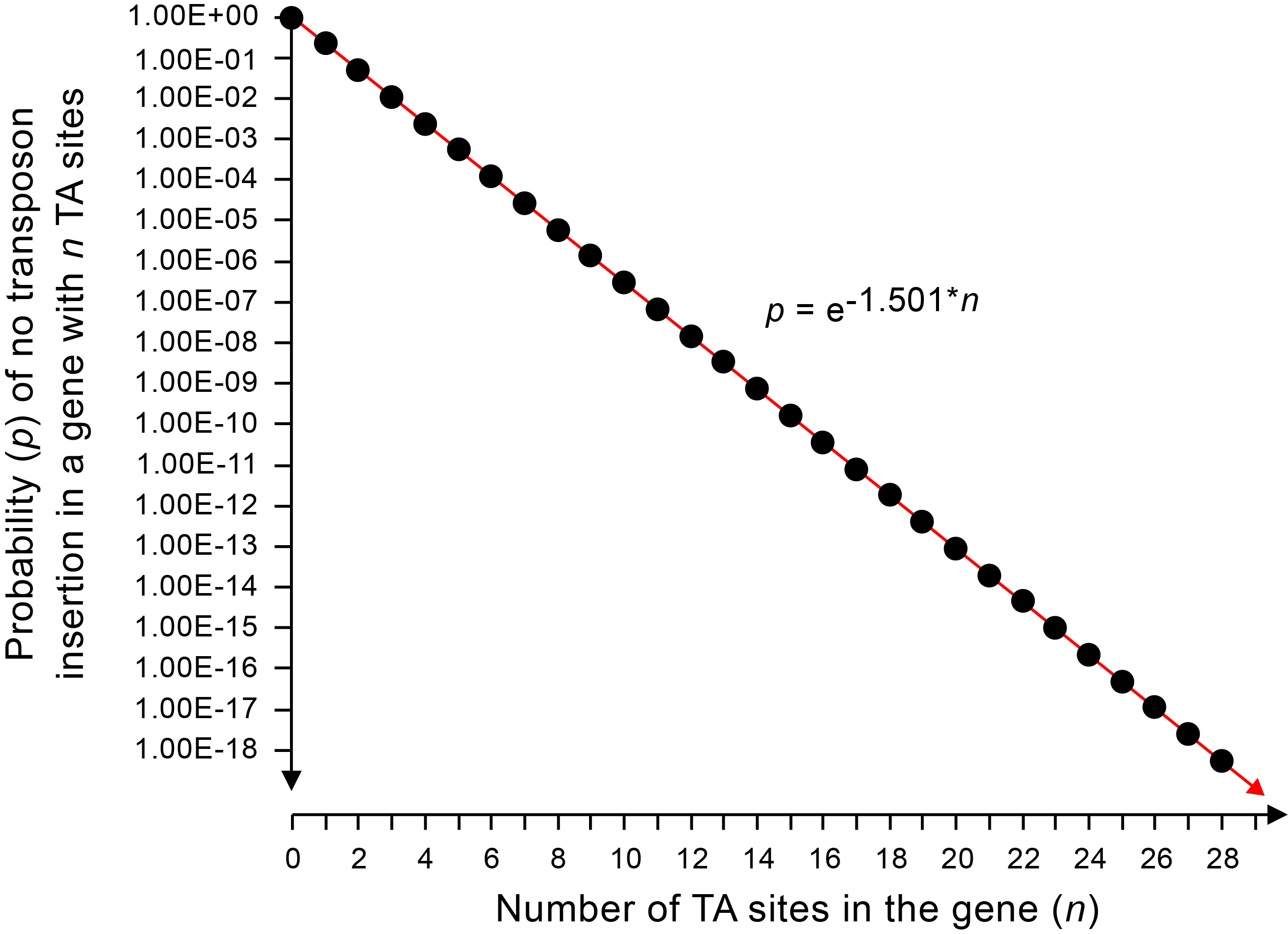

Supplement: Supplementary file 1 [file pathogens-12-00086-s001.zip › Figure S1_Janisch et al.tif]

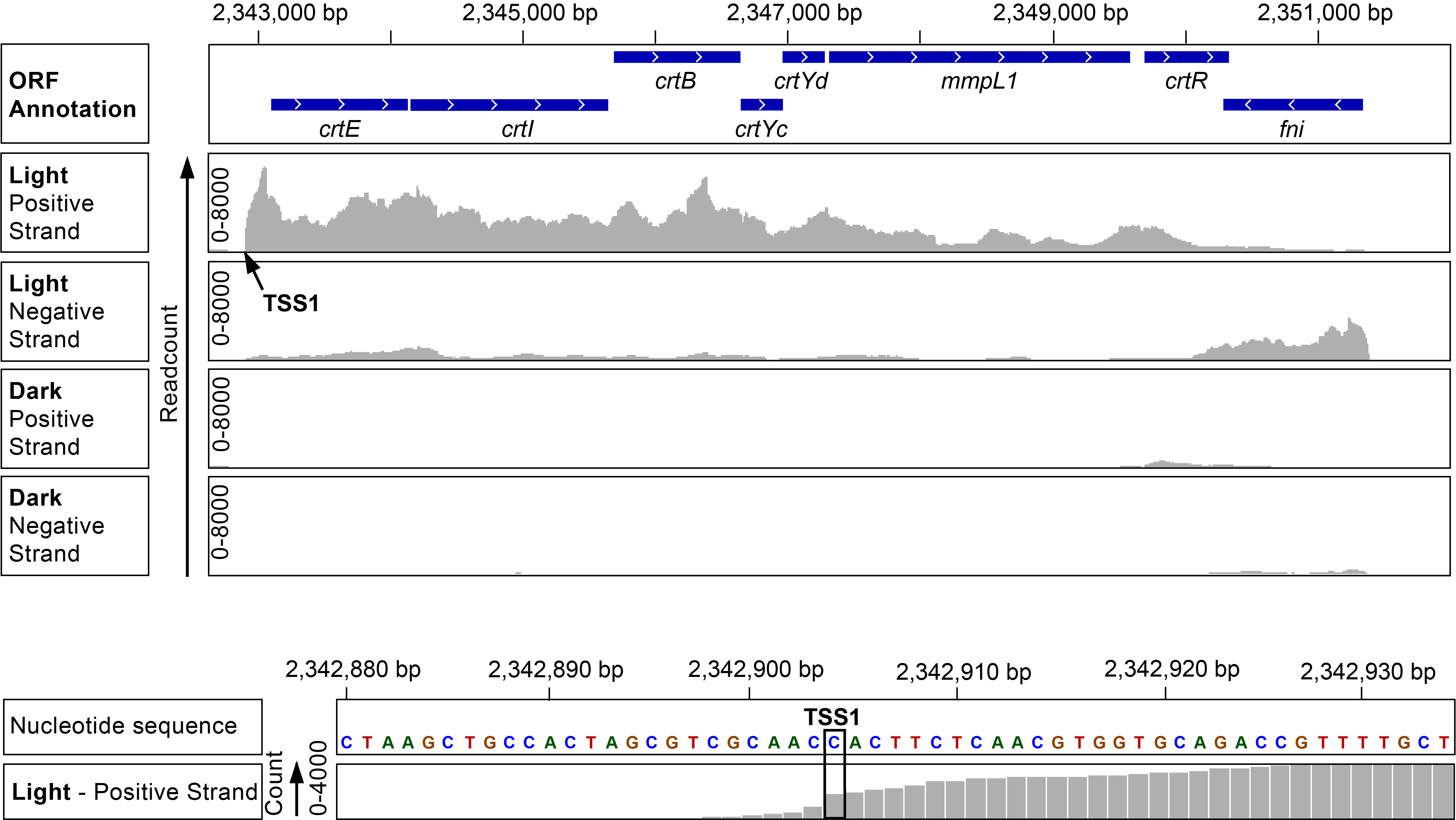

Supplement: Supplementary file 1 [file pathogens-12-00086-s001.zip › Figure S2a_Janisch et al.tif]

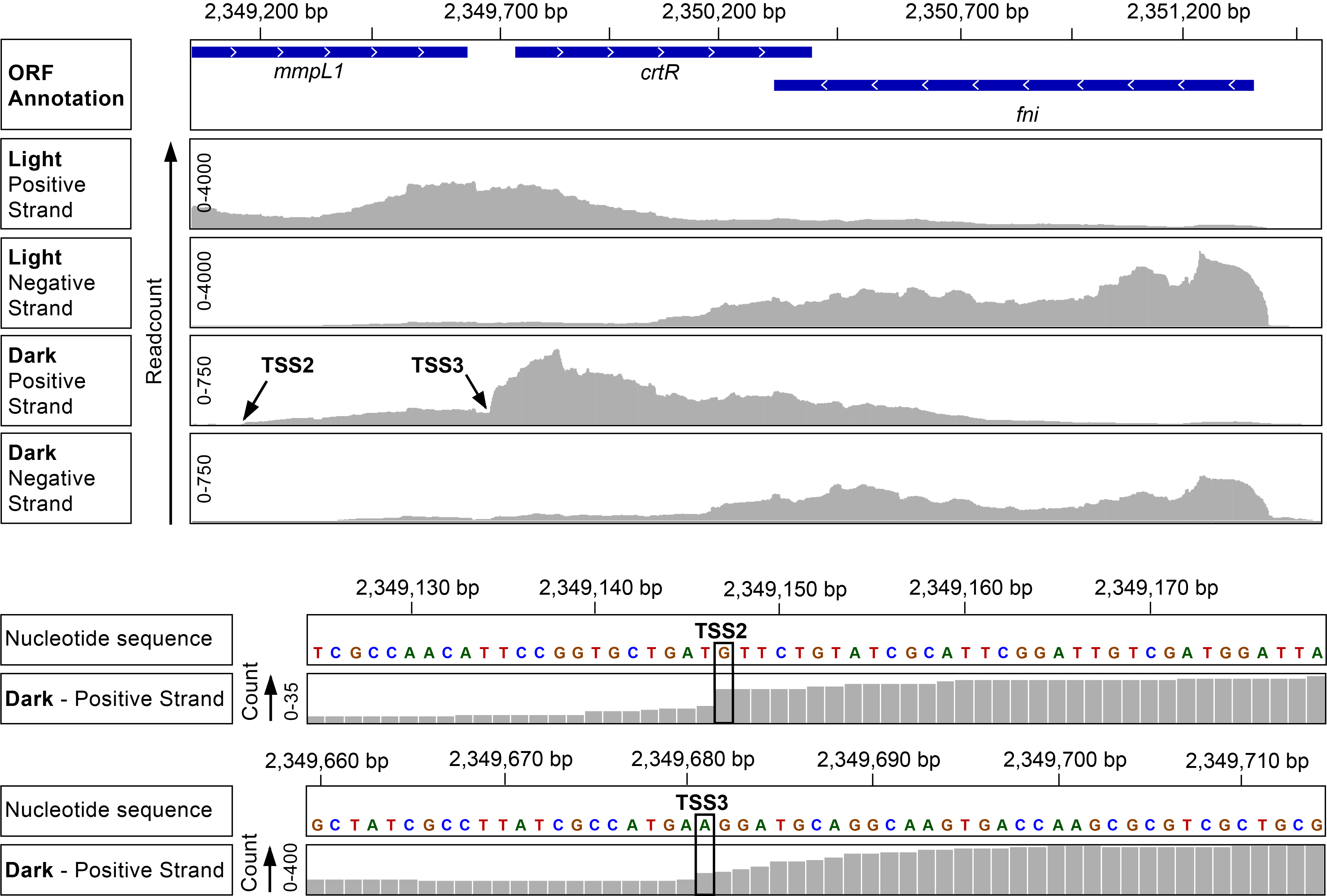

Supplement: Supplementary file 1 [file pathogens-12-00086-s001.zip › Figure S2b_Janisch et al.tif]

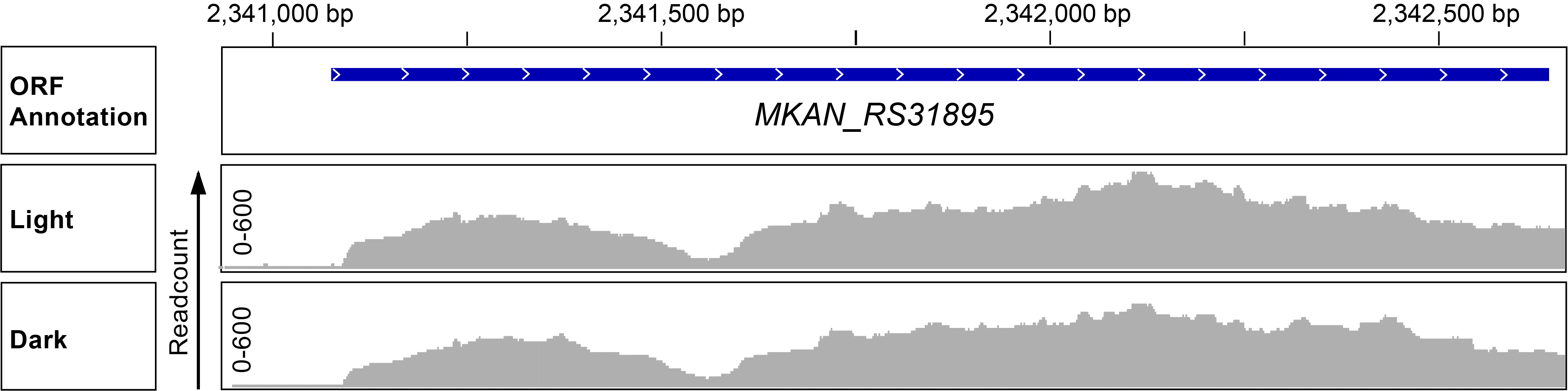

Supplement: Supplementary file 1 [file pathogens-12-00086-s001.zip › Figure S2c_Janisch et al.tif]

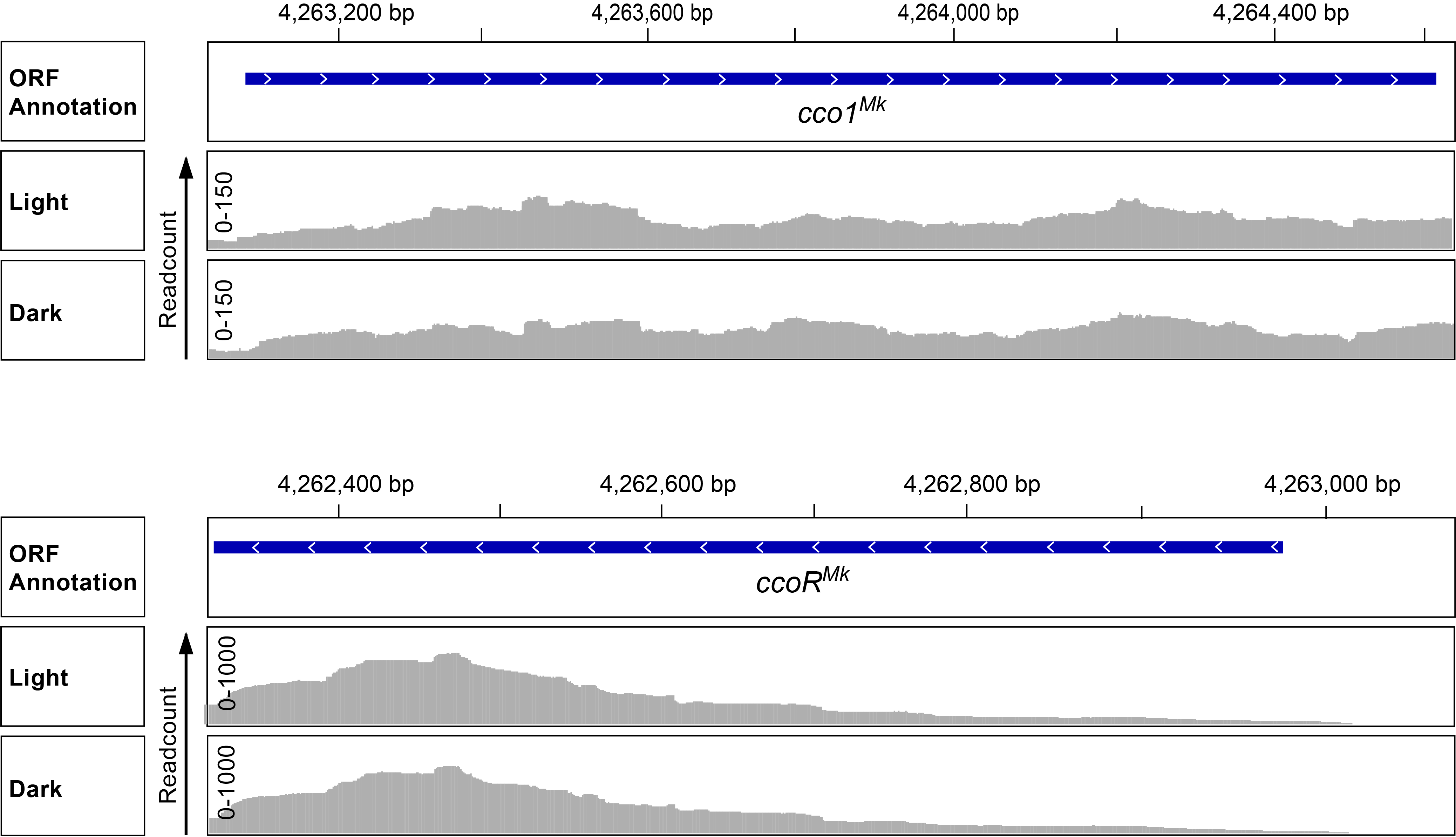

Supplement: Supplementary file 1 [file pathogens-12-00086-s001.zip › Figure S2d_Janisch et al.tif]

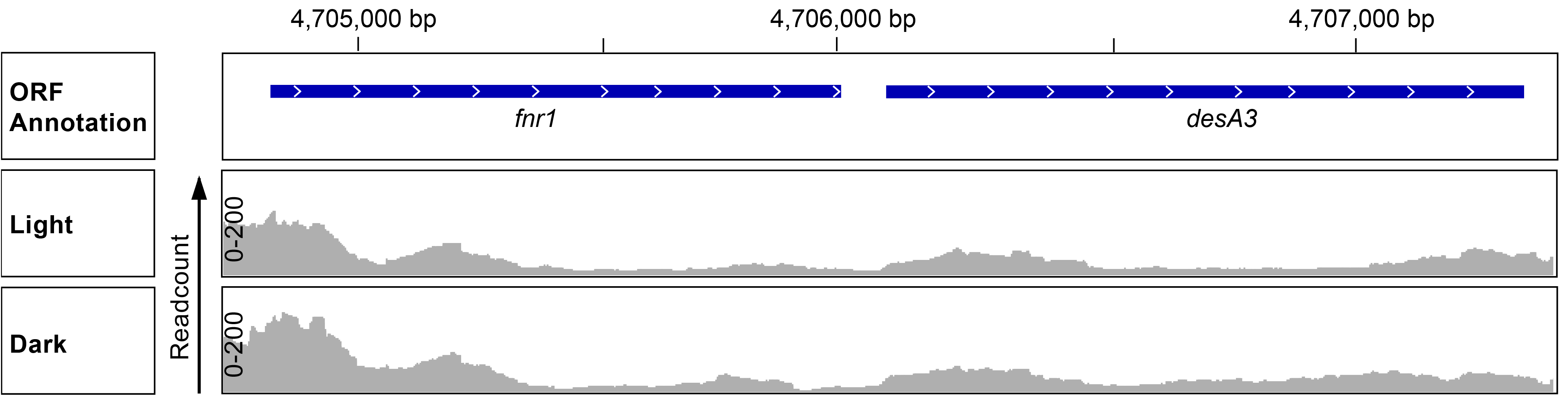

Supplement: Supplementary file 1 [file pathogens-12-00086-s001.zip › Figure S2e_Janisch et al.tif]

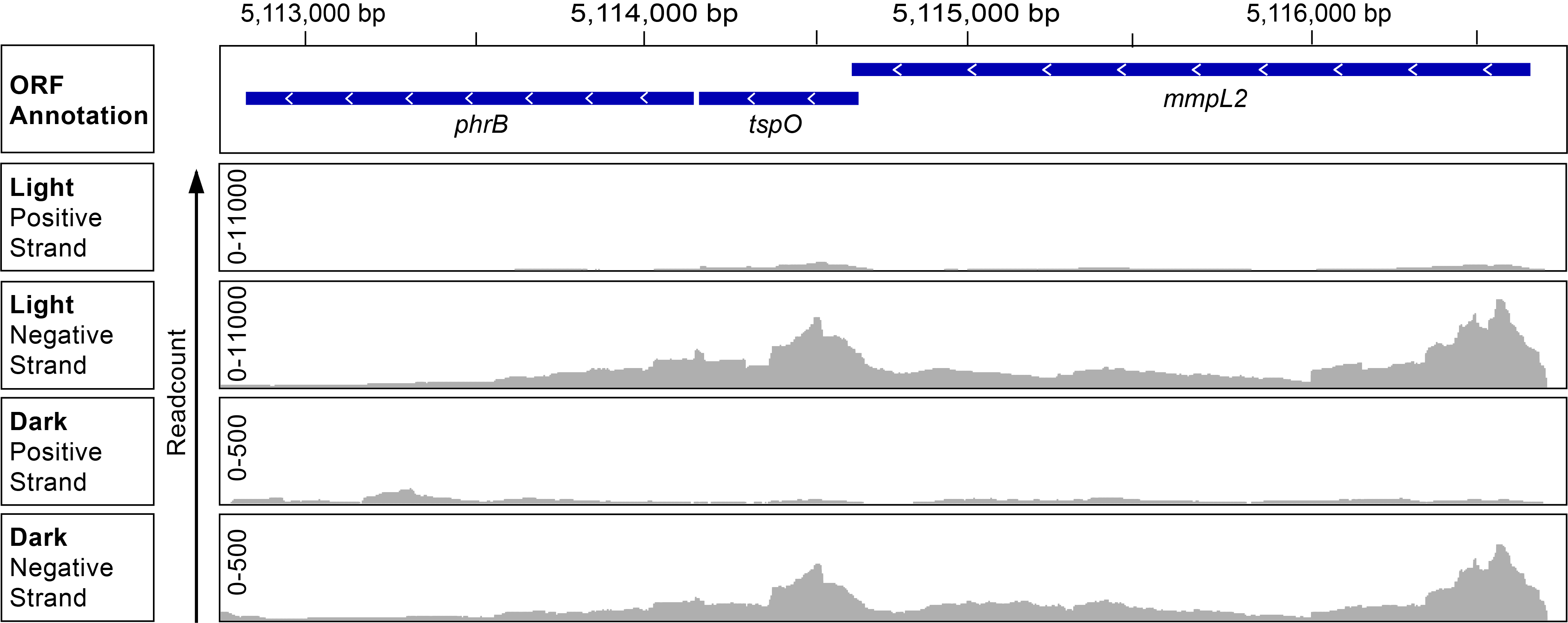

Supplement: Supplementary file 1 [file pathogens-12-00086-s001.zip › Figure S2f_Janisch et al.tif]

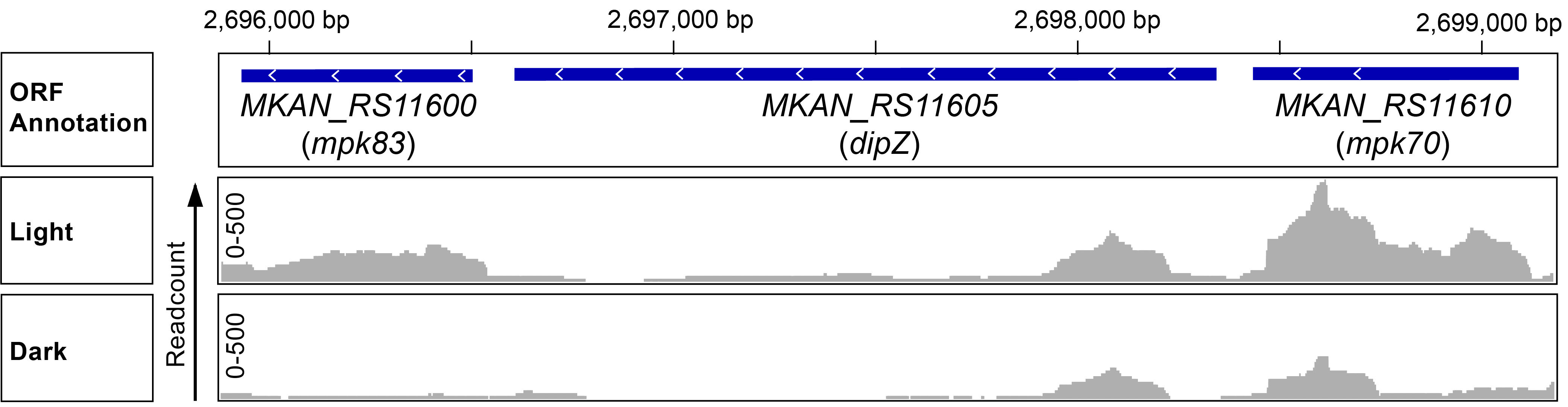

Supplement: Supplementary file 1 [file pathogens-12-00086-s001.zip › Figure S2g_Janisch et al.tif]

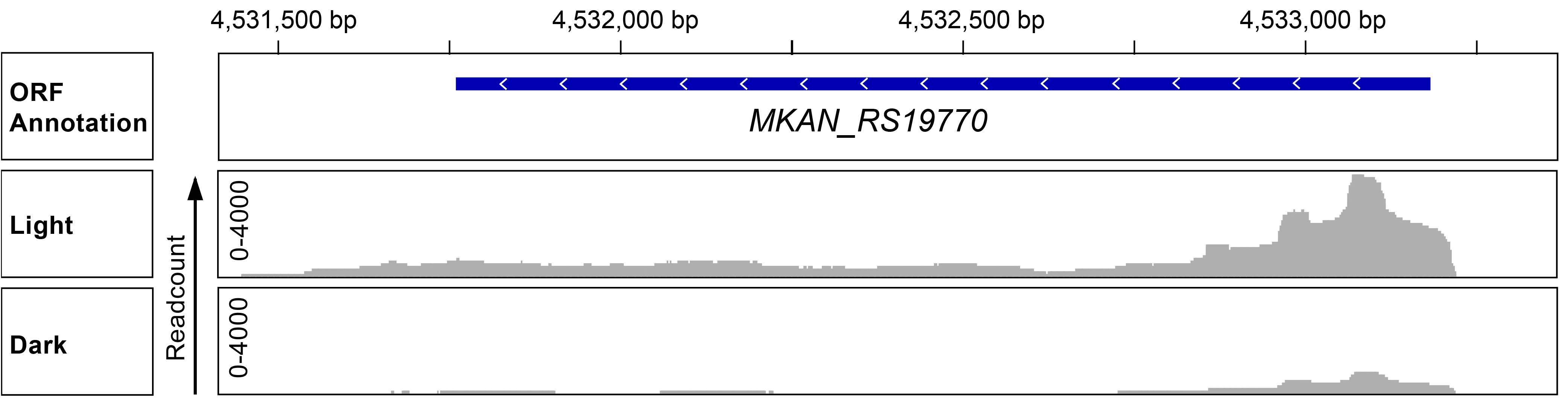

Supplement: Supplementary file 1 [file pathogens-12-00086-s001.zip › Figure S2h_Janisch et al.tif]

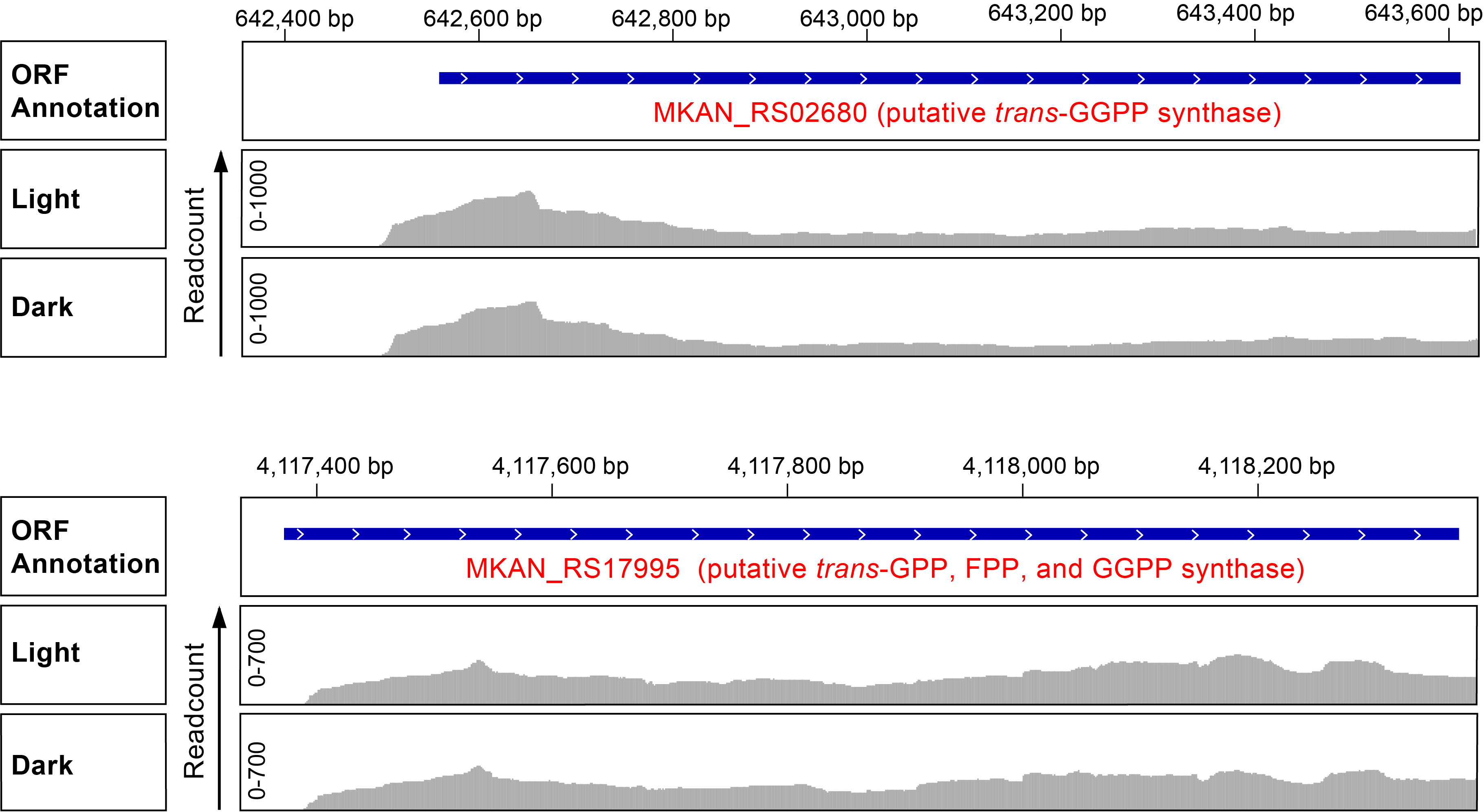

Supplement: Supplementary file 1 [file pathogens-12-00086-s001.zip › Figure S2i_Janisch et al.tif]

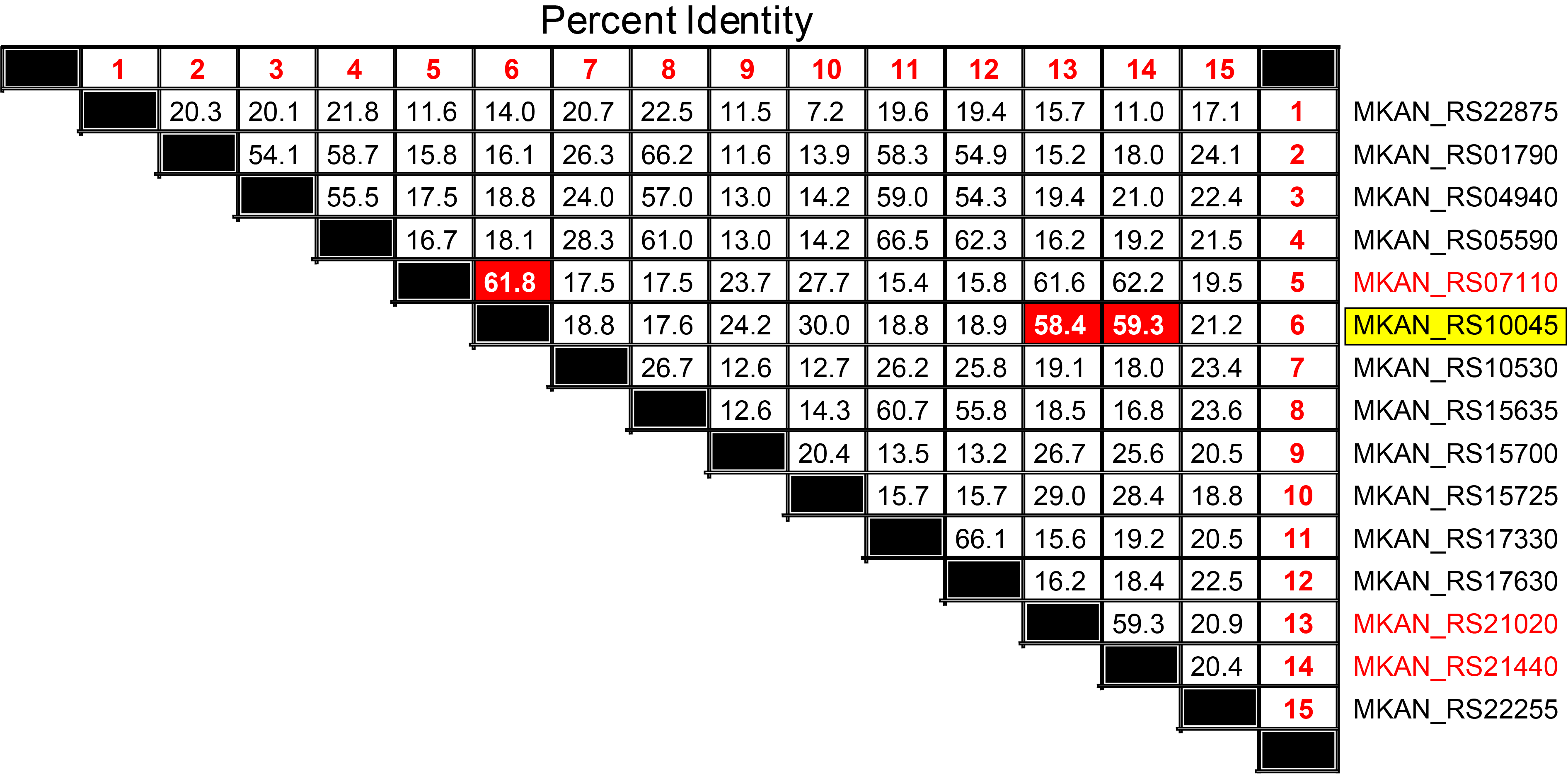

Supplement: Supplementary file 1 [file pathogens-12-00086-s001.zip › Figure S3_Janisch et al.tif]

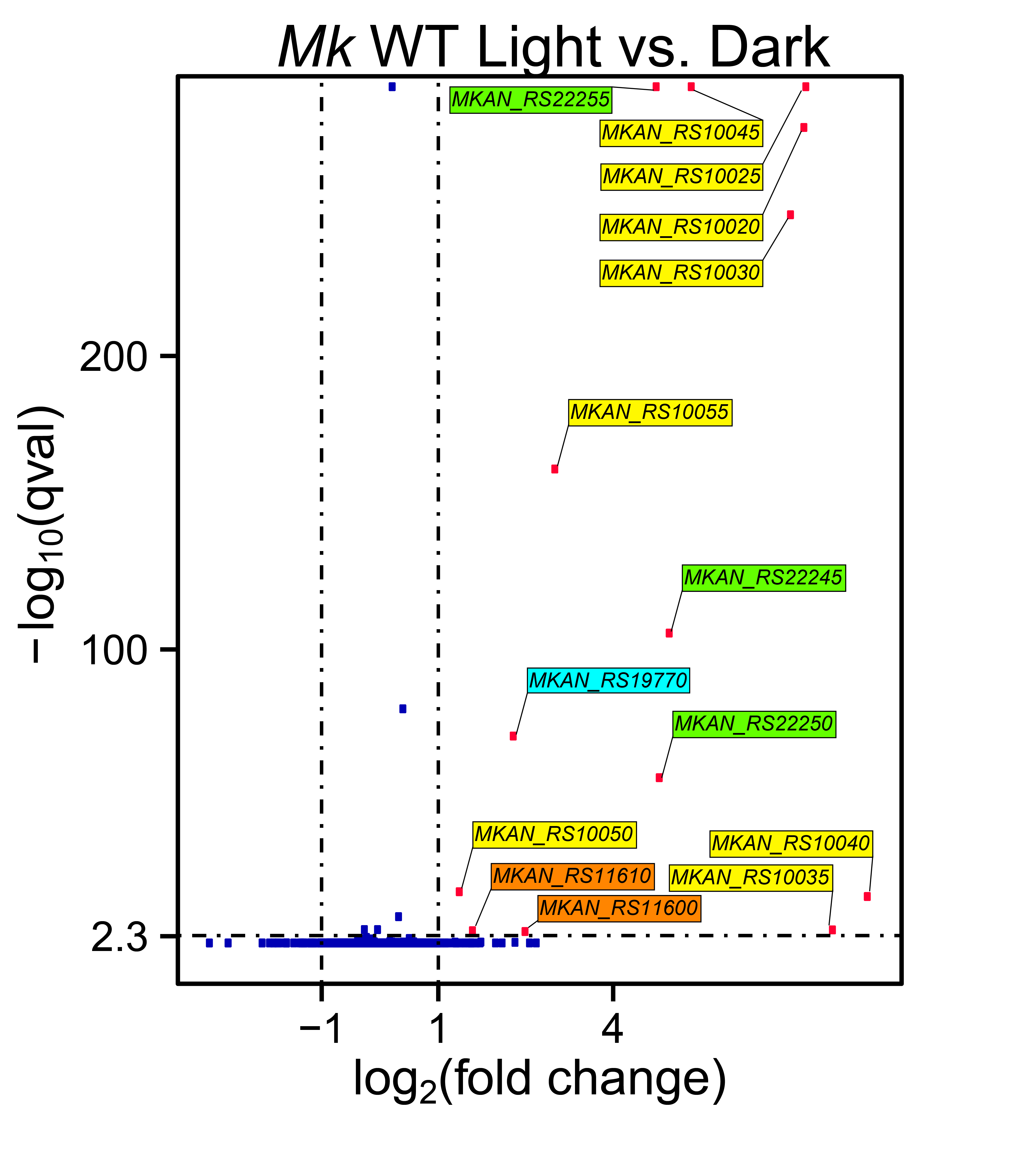

Supplement: Supplementary file 1 [file pathogens-12-00086-s001.zip › Figure S6_Janisch et al.tif]

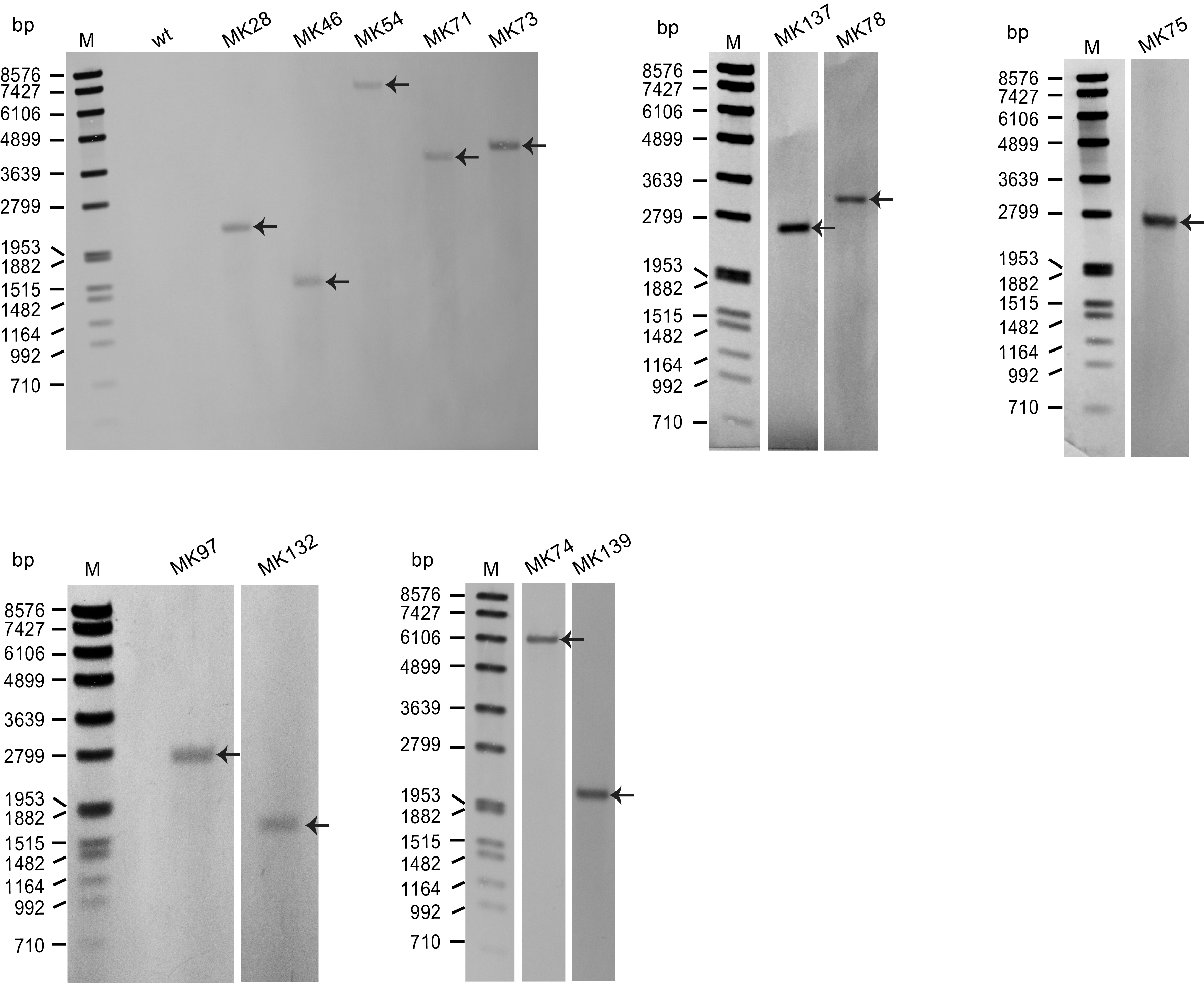

Supplement: Supplementary file 1 [file pathogens-12-00086-s001.zip › Figure S7_Janisch et al.tif]
